# The Role of Rehabilitative Ultrasound Imaging Technique in the Lumbopelvic Region as a Diagnosis and Treatment Tool in Physiotherapy: Systematic Review, Meta-Analysis and Meta-Regression

**DOI:** 10.3390/jcm10235699

**Published:** 2021-12-03

**Authors:** Samuel Fernández-Carnero, Carlos Martin-Saborido, Alexander Achalandabaso Ochoa-Ruiz de Mendoza, Alejandro Ferragut-Garcias, Juan Nicolás Cuenca-Zaldivar, Alejandro Leal-Quiñones, Cesar Calvo-Lobo, Tomas Gallego-Izquierdo

**Affiliations:** 1 Physiotherapy and Pain Group, Department of Physiotherapy and Nursing, Alcalá University, 28801 Alcalá de Henares, Spain; samuelfernandezcarnero@gmail.com (S.F.-C.); aaochoa@ujaen.es (A.A.O.-R.d.M.); alejandro_sft@yahoo.es (A.F.-G.); tomas.gallego@uah.es (T.G.-I.); 2 National School of Health, Instituto de Salud Carlos III, 28029 Madrid, Spain; 3 Área de Fisioterapia, Departamento de Ciencias de la Salud, Universidad de Jaén, Jaén, 23071 Andalucía, Spain; 4 Departamento de Enfermería y Fisioterapia, Islas Baleares University, 07122 Ciudad de Palma, Spain; 5 Rehabilitation Service, Guadarrama Hospital, 28440 Madrid, Spain; jcuenzal@yahoo.es; 6 Department of Physical Therapy, Universidad Francisco de Vitoria, 28223 Madrid, Spain; a.leal@ufv.es; 7 Faculty of Nursing, Physiotherapy and Podiatry, Universidad Complutense de Madrid, 28040 Madrid, Spain; cescalvo@ucm.es

**Keywords:** rehabilitative ultrasound imaging, real time ultrasound imaging, lumbar region, abdominal wall, pelvic floor

## Abstract

Rehabilitative ultrasound imaging (RUSI) technique seems to be a valid and reliable tool for diagnosis and treatment in physiotherapy and has been widely studied in the lumbopelvic region the last three decades. The aims for this utility in clinical settings must be review through a systematic review, meta-analysis and meta-regression. A systematic review was designed following the PRISMA (Preferred Reporting Items for Systematic Reviews and Meta-Analyses) guidelines with PROSPERO registration and per review in all phases of the process using COVIDENCE, analysis of risk of bias and meta-analysis using REVMAN, and meta-regression calculation using STATA. Database screening provided 6544 references, out of which 321 reported narrative synthesis, and 21 reported quantitative synthesis, while only 7 of them provided comparable data to meta-analyze the variables pain and muscle thickness. In most cases, the forest plots showed considerable I^2^ heterogeneity indexes for multifidus muscle thickness (I^2^ = 95%), low back pain (I^2^ = 92%) and abdominal pain (I^2^ = 95%), not important for transversus abdominis muscle thickness (I^2^ = 22%), significant heterogenity (I^2^ = 69%) depending on the subgroup and not important internal oblique muscle thickness (I^2^ = 0%) and external oblique muscle thickness (I^2^ = 0%). Meta-regression did not provide significant data for the correlations between the variables analyzed and the intervention, age, and BMI (Body Mass Index). This review reveals that RUSI could contribute to a high reliability of the measurements in the lumbopelvic region with validity and reliability for the assessments, as well as showing promising results for diagnosis and intervention assessment in physiotherapy compared to the traditional model, allowing for future lines of research in this area.

## 1. Introduction

### 1.1. Rationale

The use of an imaging diagnostic tool such as ultrasound (US) in physiotherapy became evident during the first Rehabilitative Ultrasound Imaging [1] (RUSI) Symposium, where an international consensus was reached that meant a turning point in the field, since it became published and recognized by the World Physiotherapy [2]. The second edition of this Symposium widened the conceptual framework of the US in physiotherapy and its uses, becoming evident in two publications that describe thoroughly both its scientific event program and its field of interest and competences [3,4].

The objective of using RUSI is to evaluate the activity of musculoskeletal tissue, after Dr. Stokes and Dr. Young started providing evidence by examining transverse section areas to reveal the most precise anthropometric determination [5,6,7]. Consequent evidence showed the importance of these assessments by relating volume, thickness and shape to pain and/or dysfunction [8,9], and the relevance it may have at a clinical level and to monitor the evolution of the patient.

There is a high incidence of musculoskeletal pain, being the second cause of disability [10] worldwide. The high incidence of low back pain in the world population [11,12,13] is also well known, where 80% suffers low back pain at least once in their lifetime, becoming a high reason for consultation in physiotherapy. Treatment is costly for healthcare systems, with a direct yearly repercussion of $1.02 billion [14] in Australia in 1997 and 11 billion pounds [15] in the UK in 1998.

Diagnostic imaging tools of a higher level such as magnetic resonance imaging (MRI) have shown not to be directly associated to pain generation, even among healthy people [16] because it cannot evaluate during muscle activation. Evidence of bias in the assessment with MRI [17] has also been found, which offers a new paradigm in physiotherapy work and also demonstrated the no relation of MRI injury evidenced with the symptoms, even in a 17 years follow-up study [18]. These reasons, together with the high cost of MRIs and the possibility to work at real time for muscle assessment, give an opportunity for ultrasound.

RUSI seems to be a high-level validation technique, since different comparisons have been carried out in each of the areas of interest—lumbar [19], abdominal [20,21] and pelvic floor [22,23]—and in all cases the intra-class correlation coefficients were higher or equal to 0.8. Therefore, it could be very interesting to use RUSI at a clinical level.

To date, several reviews on the RUSI technique in the lumbopelvic region [24,25,26,27,28,29] have been carried out, but never focused on the objectives described in this systematic review.

These reviews have dealt with the evaluation of paraspinal characteristics and conditions including ligaments and muscle tension [26]. Additionally, the evaluation and treatment of the transversus abdominis and multifidus muscles in patients with low back pain [27], analyzing the ADIM maneuver as a means to detect muscle dysfunction and the ultrasound as a tool to measure it, show the validity and confidence of the method. Another study evaluated the effectiveness of real-time ultrasound as a tool in biofeedback for muscle training [29], identifying the term “RTUS” as meaning the use of real-time ultrasound to evaluate movement and as biofeedback. Related to the pelvic floor (although not systematic due to the type and quality of the published evidence), in patients with urinary incontinence and/or prolapse [28], the validity of RUSI in the quantitative evaluation of abdominal and lumbar muscles and on the validity of measurements and activation during submaximal isometric contractions [29,30] conclude in favor of the validity of this ultrasound technique for submaximal isometric contractions.

### 1.2. Objectives

Based on the previous pilot study published [30], to ensure the concordance between reviewers, this study aims to evaluate the RUSI technique in order to answer the following questions: Is the RUSI technique a reliable diagnostic and treatment tool that offers validity and reliability in physiotherapy? Does it offer an advantage in the treatment with biofeedback, and as an assessment method of the intervention performed on patients?

## 2. Materials and Methods

A systematic review was designed in accordance with the Preferred Reporting Items for Systematic Reviews and Meta-Analyses guidelines. The Cochrane Collaboration guidelines [31,32] were also used (study selection, eligibility criteria application, data extraction, and statistical analysis).

### 2.1. Eligibility Criteria and Information Sources

Several inclusion criteria were considered: (1) randomized clinical trials or controlled prospective designs; (2) studies which contain the sonograph as an assessment tool (in morphology and behavior muscle view) and the treatment (biofeedback tool) of the lumbopelvic region; (3) randomized clinical trials which compare MRI/electromyography (EMG) versus US; (4) validity and reliability studies and quantitative and/or reliability of lumbopelvic and abdominal region studies about sonography and the lumbopelvic and abdominal region; (5) evaluation studies about US education, operation and interpretation; (6) adults > 18 years with and without lumbopelvic pain. Additionally, exclusion criteria: (1) non-randomized studies; (2) US interventional medical purpose (tissue injuries) tumors, tears, inflammatory disease, etc.; (3) no abstract available or incomplete; (4) abstracts from Congress, Symposium, etc.; (5) pediatric population.

### 2.2. Search Strategy

A search strategy with free and controlled terms about the lumbopelvic region was established (see Supplementary File S1 available online) for the full search strategy with detailed database information accessed and peer review assessment. The PRISMA (Preferred Reporting Items for Systematic Reviews and Meta-Analyses) [32] recommendations were followed to elaborate this review.

### 2.3. Selection Process and Data Collection

Once the files (.ris) had been extracted from all the databases, they were exported to the specific program COVIDENCE [33] and duplicates were detected. The reviewers were blinded to each other’s opinions in the three phases that this tool offers (title and abstract screening, full text screening, and extraction) and one of them resolved all possible conflicts after peer-review screening. Inclusion criteria for the reviewers to accept the studies were established. Data were extracted by one reviewer and checked by others using customized forms.

### 2.4. Data Items

The variables that could be extracted were muscle thickness and pain, and the intervention was motor control exercises.

### 2.5. Study Risk of Bias Assessment

Once the studies were screened, they were exported to the REVMAN (Version 5.3. Copenhagen: The Nordic Cochrane Centre, The Cochrane Collaboration, 2014) [34] tool to carry out the risk of bias analysis recommended by the Cochrane Manual [31] (chapter 8.3): Risk of Bias Tool. The risk of bias tool evaluates seven domains (see Supplementary File S2 available online). Rather than a scale, it is a verification tool that evaluates the risk of bias of each of the domains (selection, performance, detection, attrition, reporting, and other biases). To that aim, two reviewers assessed the risk of bias and performed the data extraction for meta-analysis. The tool itself generates a graph with colors: red (high-risk), orange (unclear-risk), and green (low-risk).

### 2.6. Synthesis Methods

The averages of the variables to be compared were extracted with an inverse-variance statistical calculation based on the inverse variance through a fixed-effects model, which suggested a homogeneity hypothesis between effect sizes. The result provided the level of heterogeneity (I^2^), considering that: 0–40% may not be important; 30–60% may represent moderate heterogeneity; 50–90% may represent significant heterogeneity; and 75–100% may represent considerable heterogeneity.

Secondarily, a meta-regression was carried out using the STATA program (StataCorp. 2015. Stata Statistical Software: Release 14. College Station, TX: StataCorp LP) [35], in order to explain the data obtained in the meta-analyses.

## 3. Results

### 3.1. Study Selection (Flow of Studies)

**Figure 1:**
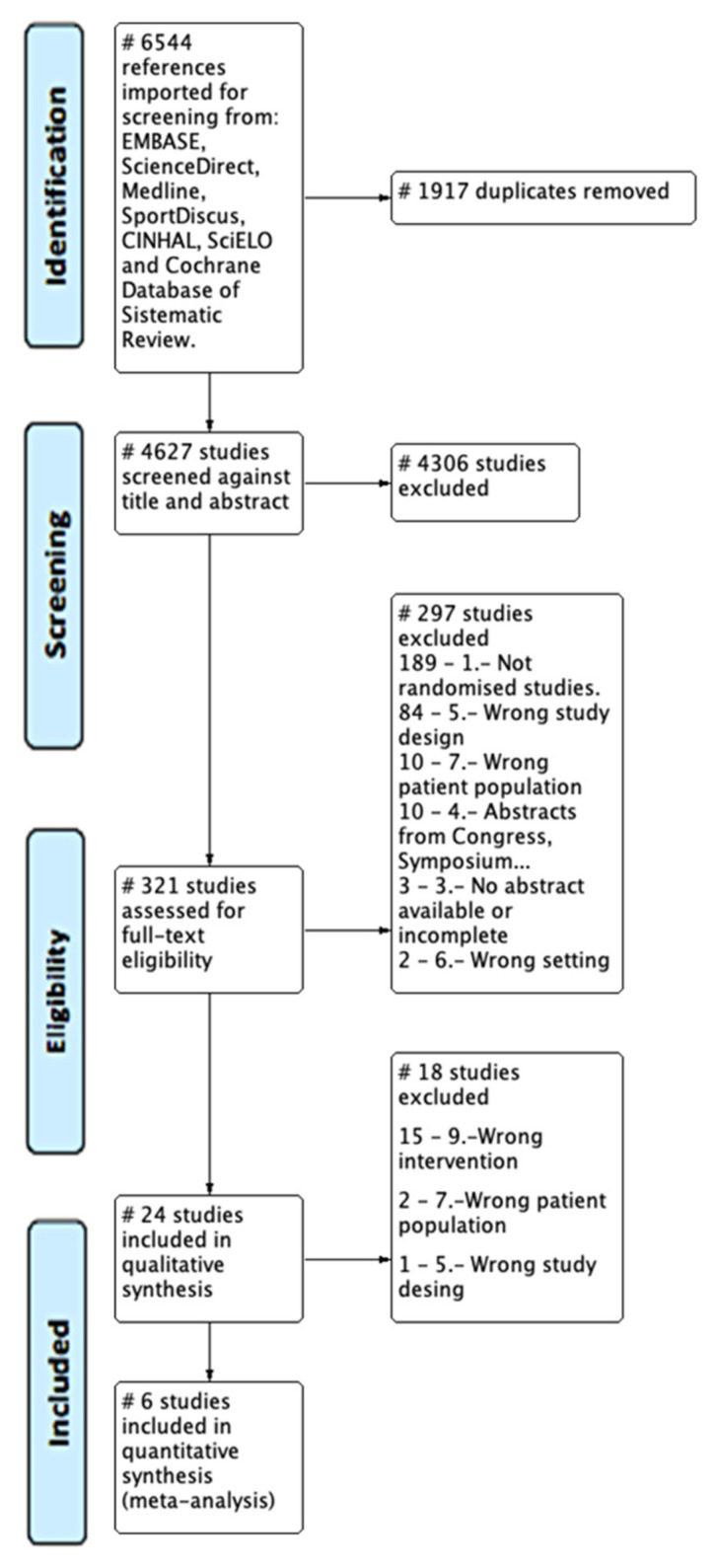
PRISMA flowchart.

The databases analyzed provided 6544 references, out of which 1917 were duplicates, resulting in 4627 references for the “title and abstract” phase. In this phase, 4306 references were dismissed, resulting in a total of 321. In the “full text review” phase, 296 were dismissed and finally, 24 references were included for the qualitative synthesis. The references of this last phase were analyzed and 6 were selected for the quantitative synthesis (Figure 1 PRISMA flowchart).

### 3.2. Study Characteristics and Risk of Bias

The risk of bias analysis of each of these articles was performed with the RevMan tool (see Supplementary File S2 for Risk of Bias REVMAN tool), and the Risk of Bias tables with evidence were extracted in the REVMAN tool (see Supplementary File S3 for Risk of Bias REVMAN tool). The summary of the characteristics of studies included according to regions resulted in the following:

#### 3.2.1. Lumbar

The lumbar region in Supplementary File S2 (Graphs S1 and S2) provided a low risk of bias for Hebert et al. [36]. However, the rest of the authors found a high or unclear risk. It is important to highlight that few studies showed a high risk of bias, and that was due to bad planning in the methodology. The study with the highest risk of bias was that of Van et al. [37], in which neither the participants, the staff, nor the statistician were blinded.

#### 3.2.2. Abdominal

The risk of bias assessment for the studies of the abdominal region was high in some cases. Three of the fifteen studies analyzed had a low risk of bias (Ferreira et al. [38], Guthrie et al. [39] and Teyhen et al. [40]) in Supplementary File S2 (Graphs S3 and S4). The greatest bias in the abdominal region studies was related to group allocation and blinding of participants, evaluators, and statisticians.

Blinding of patients may have been the biggest drawback, since it is difficult to blind an intervention in physiotherapy for the subjects being researched.

#### 3.2.3. Pelvic Floor

Few randomized studies have been published about the pelvic floor region that could be screened for bias analysis. None of the four studies that made it to this phase complied fully with the seven domains of the RevMan tool in Supplementary File S2 (Graphs S5 and S6), and the study by Bernardes et al. [41] does not clearly explain the blinding process.

The summary of the studies analyzed in this phase regarding author, design, population, statisticians, and intervention (see Table 1) showed that certain studies were not comparable due to differences in the type of intervention, population or design. Therefore, only six studies were finally used for the quantitative synthesis (see Table 2).

### 3.3. Results of Syntheses

The comparable variables in the lumbar and abdominal regions were pain and muscle thickness in patients treated with exercise, and the complete data from meta-analysis were collected (see Supplementary Material S4: Complete annotated forest plot-graphs and tables).

Data summaries of the variables described for the lumbar region for meta-analysis are shown in Supplementary File S4 (Tables S3 and S4). The result for the variable muscle thickness of lumbar multifidus was in favor of the control group, Supplementary File S4 (Graph S7) with a *p* < 0.0001, chi^2^ = 18.78, and high heterogeneity of I^2^ = 95%.

Meta-analysis of the variable pain in the lumbar region showed a result in favor of exercise Supplementary File S4 (Graph S8), with a *p* = 0.21 and chi^2^ = 1.55, and moderate-low heterogeneity of I^2^ = 36%, but a CI (−0.93, −0.53) in favor of the exercise group. Motor control exercise seems to be more beneficial to low back pain as the authors have been reported using motor control exercise strategies.

Data summaries of the abdominal variables muscle thickness and pain, for meta-analysis, are shown in Supplementary File S4 (Tables S5–S8). The table for transverse abdominis (TrA) muscle thickness was structured in two subgroups: thickness change and thickness average.

Meta-analysis of the variable pain in the abdominal region resulted in favor of the control group Supplementary File S4 (Graph S9), with a *p* < 0.00001 and chi^2^ = 37.31, and heterogeneity was high, at I^2^ = 92%.

For meta-analysis of the variable thickness of the TrA, a subgroup analysis was necessary, due to the results being presented in different ways, according to the methodology of the studies. In the case of Ferreira et al. [38] and Halliday et al. [42], data were provided of the contraction of the TrA muscle (contraction ratio or %). However, the studies by Navabi et al. [43] and Shamsi et al. [44] provided data of the average TrA muscle thickness. The result was in favor of exercise Supplementary File S4 (Graph S10), although relatively close to the line of no effect. The subgroup values expressed in % obtained a *p* = 0.26 for a chi^2^ = 1.28 and a heterogeneity of I^2^ = 22%, considered low. The subgroup whose values were expressed in muscle thickness average obtained a *p* = 0.07 for a chi^2^ = 3.20 and a moderate heterogeneity of I^2^ = 69%. Lastly, the global values of both subgroups had a *p* = 0.0001 for a chi^2^ = 16.23 and a high level of heterogeneity, at I^2^ = 86%.

Meta-analysis of the variable thickness of the internal oblique muscle obtained a result in the line of no effect Supplementary File S4 (Graph S11), with a *p* = 0.92 for a chi^2^ = 0.01 and low heterogeneity, at I^2^ = 0%.

In the analysis of the variable thickness of the external oblique muscle, the result was neither in favor nor against the intervention Supplementary File S4 (Graph S12), although this seems logical due to the non-existent implication of this muscle in the activities evaluated; *p* = 0.44 for a chi^2^ = 0.61 and low heterogeneity of I^2^ = 0%.

Given the high heterogeneity found, meta-regression was used to explore it. The variables multifidus muscle thickness, TrA muscle thickness and pain were compared with meta-regression in relation to the BMI, age and length of the intervention.

Data from four of the studies were extracted (Ferreira et al. 2010, Navabi et al. 2017, Shamsi et al. 2016 and Halliday et al. 2016) for the variables pain and muscle thickness in relation to age and length of the intervention Supplementary File S4 (Tables S9 and S10). Data results provided a *p* > 0.05; hence, length of the intervention and age were not statistically significant for abdominal pain and muscle thickness.

## 4. Discussion

Out of the 321 articles found, only 6 met the criteria to be compared and discussion about these records must be highlighted.

The results obtained from the meta-analyses conclude that motor control exercises are more beneficial for certain variables, such as low back pain (chi^2^ = 1.55 and heterogeneity I^2^ = 36%) or for TrA muscle thickness (chi^2^ = 1.28 and heterogeneity I^2^ = 22%). In these cases, heterogeneity levels are low; however, for the remaining variables (multifidus muscles thickness, abdominal pain, internal oblique muscle and external oblique muscle), the meta-analyses results were in favor of the control group or with high heterogeneity indexes, which may be due to the small number of studies compared, since their methodology prevented them from being included in the meta-analysis.

Subsequently, a meta-regression was carried out for the abdominal and lumbar region studies meta-analyzed, and determined that there is no relationship for age or length of the intervention in the variables pain and muscle thickness. Some limitations were found (i.e., some studies presented data of the right side, some of the left side, some in terms of muscle thickness averages, others as contraction percentage, etc.) which, if avoided, may have provided different results.

The use of US for the lumbar region is a valid method for muscle assessment of the low back region in comparison to MRI [45,46], and the transducer has become a reliable tool for everyday use. The results demonstrated high correlation (ICC = 0.91–0.97), US-MRI correlation (r = 0.75–0.93) for muscle thickness and (r = 0.63–0.82) for the cross-sectional area (CSA). Within this technique, morphology assessment may be decisive. For the lumbar region, the studies by Stokes et al. [47] and Hides et al., [48] on the characteristics of the lumbar wall can be highlighted, as well as the reference data analyzed in the study by Hides with 120 healthy subjects, resulting in three morphologies where the population could be framed. In this validation process, a very interesting study is the one by Koppenhaver et al. [49] that uses repeated measures to find out the standard error, in order to discover the number of measurements needed to decrease error bias. Thus, both in the TrA and in the lumbar multifidus muscle, error decreases by 20% when two measurements are done, by 50% with three measurements, and precision increases, though only slightly, with further measurements.

Validity and reliability between expert and novice were found [50], with intra- and inter-rater correlation indexes of 97–99% for the L4–5 region, and slightly lower for the L2–3 region. This situation was evaluated in subsequent studies, with similar results [51].

Afterwards, studies began to be published that assess the contraction fraction by calculating the percentage of thickness change [49,51,52,53,54], concluding that precision in inter-rater measurements is more optimal when three consecutive measurements are performed.

Additionally, this tool became more frequently used for different activities, since its clinical use needed validity. Different maneuvers were tested, such as maximum isometric contraction in extension [19], and distinguishing between deep and superficial multifidus muscles, with a reliability value of ICC = 0.84–1.00. The prone, supine and supine crook-lying positions were also evaluated for the CAL [55] (contralateral arm lift), concluding the prone the best. In this line, prone and side-lying were also compared [56], finding high correlation for CSA of multifidus muscles; moreover, the evaluation between prone and standing positions showed no significant differences. We also know that the standing position shows less thickness than standing with hyperlordosis, and, likewise, the thickness of the TrA is greater when sitting upright than when stooping forward [57], so these positions must be considered when treating patients with low back pain.

The correlation between US and EMG was also found, in order to resolve the concordance between both tools. Kim et al. [58] reported a value of r = 0.51–0.61, and Kiesel et al. [59] a value of r = 0.79, *p* < 0.001. These results could be potentially useful for clinical use in patient feedback and as a diagnosis for clinical decisions.

Another criterion of great interest for patient assessment lies on the analysis of the CSA [60,61,62,63], which has shown different correlations between pain and CSA, but all of them converging in the same clinical applicability. Huang et al. [61] evaluated the CSA and the CSA ratio by performing a correlation between measurements and linear regression, concluding that the CSA was larger in the unaffected side than in the affected one, and the ratio of both sides would be 1.16 ± 0.10. Hides et al. [62] evaluated professional football players, obtaining similar results, since players with low back pain had significantly smaller CSA at the start of the season than those of players without pain, and their CSAs also increased more significantly. Regarding muscle activation, there were no significant differences (*p* > 0.005). Wallwork et al. [63] demonstrated a smaller CSA of the multifidus muscle in patients with lumbar pain as compared to healthy ones (*p* = 0.001) and a smaller percentage of contraction in pathological versus healthy subjects (*p* = 0.02).

In one way or another, the existence of pain has come to show that it produces inhibition of the contraction or changes in the morphology of the CSA. A study with induced longissimus [64] muscle pain showed a value of *p* > 0.01 for the lumbar multifidus muscles and TrA, which proves the interaction of the lumbopelvic region as a functional unit.

The instrumentation has been studied and the difference between exploring with linear probe or convex has little differences proved by a phantom [65]. Another study [66] evaluates the CSA in the multifidus muscles with both probes, measuring the images in a post-acquisition software, resulting in an ICC = 0.78–0.99 and a statistical significance of *p* > 0.05. Along this line, the study that analyzed the orientation of the probe in three spatial axes [9] was significant, concluding that, with a similar measurement of the TrA muscle and bladder base movement, a variation of 5–10° is possible in all of the axes.

Abdominal wall US has a deep development. In asymptomatic states, the characteristics of the abdominal wall of males versus those of females present differences [67], with greater thickness of the TrA and internal oblique in men. However, there was a greater change in the contraction of the TrA with the abdominal drawing-in maneuver (ADIM). These differences are very important to bear in mind when the study population includes men and women. In line with this evidence, normality data about the abdominal wall thickness in a healthy population [68] concludes that thickness decreases in the following way: TrA < external oblique < internal oblique < rectus abdominis, and with the same result to the previous study [69].

Furthermore, the measurements of the abdominal muscle wall and perimuscular connective tissue in patients versus healthy subjects were evaluated [70], and three conclusions were reached: rectus abdominis and perimuscular connective tissue are thinner, and there is an increase of the linea alba, with exceptions detected in pregnant women [71].

The abdominal linea alba has been related to lumbopelvic pain, since it increases in these subjects. For that reason, this variable has been evaluated and compared to other assessment methods previously used. Some questioned the validity of assessing the between-recti distance using the therapist’s fingers or a “king’s foot” on the surface of the patient, but both options have been validated [72,73] and are considered reliable. However, some exceptions must be made, since the measurement of the linea alba using fingers showed between-rater and between-days reliability (the data were related to the number of fingers that fit in), but the US measurement is more specific, providing a quantitative variable. Nevertheless, the assessment was performed both at the supra and infraumbilical levels, with greater supraumbilical concordance with both techniques (ICC = 0.79, *p* < 0.0001 and ICC = 0.71, *p* < 0.0001) and no significant differences in either measuring technique (*p* > 0.05). It should also be pointed out that between-recti distance (IRD) was studied because a link was found between muscle activity and distance of the abdominal wall [74] after a six-month follow-up of post-partum women without treatment. After six months, it was confirmed that the measurements from cranial to caudal did not go back to normal—1.80 ± 0.72, 2.13 ± 0.65, 1.81 ± 0.62 and 1.16 ± 0.58 cm—and muscle activation also decreased in comparison with nulliparous women.

The muscles were also evaluated at rest and in contraction with the ADIM maneuver by physiotherapists with only eight hours of training in US versus expert physiotherapists [20], obtaining an ICC > 0.97. Both the ADIM and the ASLR [75,76] were validated given that the mobility of the hand could create a bias, and the tests themselves could be biased and conclude the normal contraction compared to patients. The ADIM maneuver has shown to be more specific for the TrA contraction evaluation than ASLR [77]. The active straight leg raise test (ASLR) has demonstrated a great difference between healthy subjects, who present greater percentage of change in thickness of the TrA (23.7%) than pathological subjects [75] (6.4%) and the internal oblique (11.2% in patients and 5.7% in healthy patients, respectively), and it proves to be an excellent test for patient exploration. At a clinical setting, forced exhalation is also interesting for patient evaluation, since significant differences exist (*p* < 0.005) in the contraction of the TrA [78] between healthy subjects and those with low back pain.

Additionally, it must be pointed out that the validated tests performed on patients were dynamic and carried out in both decubitus and standing positions by using a binding tool (belt) and by the traditional manual technique that fixes the probe to the abdominal wall [79]. This way, the traditional manual binding resulted in an ICC = 0.67–0.79 for patients and an ICC = 0.7–0.86 for healthy subjects, whereas the levels obtained with the belt were better: ICC = 0.93–0.98 for patients and ICC = 0.97–0.99 for healthy subjects.

Intra- and inter-rater validity has been constant in the analysis of abdominal wall, obtaining excellent reliability values since the beginning [20,53] till today [72,76,79], with intra/inter examiners and between-days higher than 0.8–0.9.

In addition, US has been compared to EMG [58,80,81,82,83], and the US as a contraction assessment tool shows divergences in the studies. A study carried out with healthy subjects using M-mode US and compared with fine needle EMG provided concordant results [84]. However, the study that analyzed muscle contraction with fine needle EMG against US during the ADIM and ASLR maneuvers in patients versus controls [85] presented a low relationship (ASLR, r = 0.28 ± 0.09; ADIM, r = 0.35 ± 0.11). The latest study published about US and EMG provided good to excellent [81] concordance levels for the TrA (r = 0.74, *p* < 0.001) and internal oblique (r = 0.85, *p* < 0.001). When comparing abdominal US diagnosis with surface EMG [58,83] in intra/inter examiner measurements and manual palpation for the internal oblique and the multifidus muscles in healthy subjects, a good ICC was observed (ICC = 0.81–0.98). However, palpation showed lower sensitivity (*p* < 0.01) and a moderate correlation between US and EMG (r = 0.51–0.61). The second study performs a monitorization of the TrA muscle with US, internal oblique and EMGs for the rectus abdominis, and external oblique for the ADIM maneuver with ICC = 0.77–0.95 for both groups. Therefore, the combination of these two techniques provided excellent results.

However, these data contradict the ones obtained when using EMG with fine needle [81], this time in TrA, internal oblique and external oblique, concluding that there is a correlation between EMG and US of r = 0.74 and r = 0.85 for TrA and internal oblique, respectively.

In the validity carried out on the automatic activity of abdominal muscles in healthy subjects and subjects with lumbopelvic pain [86] and comparing in supine, sitting on a 65cm ball and sitting but lifting the left foot 10cm, the results were 0.88–0.95 within-day and 0.85–0.94 between-days for the healthy group, and 0.89–0.94 within-day for the volunteers with low back pain.

In order to assess muscle contraction in the abdominal wall and to determine if the lack of activation is related to a pathology, weight bearing [87] was applied, simulated with a previously validated protocol [88]. A significant difference was found in patients with low back pain versus healthy ones (*p* < 0.0001), due to a smaller shortening of the TrA and a greater increase of the internal oblique (*p* = 0.002). No differences were found in the thickness of the TrA. This conclusion is interesting due to the type of activity, which does not require training of the patients and is based on daily movements.

A few validity studies have also been found on the pelvic floor region. The title of the study: “Can we ‘feel’ our fingers as well as we ‘see’ with ultrasound?” [89], shows the need for using the transducer in this area, where transperineal US [90] and palpation are used in patients with incontinence and puborectalis muscle avulsion. Concordance level in the assessment with imaging was K = 0.618, versus palpation, with K = 0.467. Other relevant studies are those in which pelvic floor palpation and US [91,92] are compared in order to assess contraction in patients who have been trained on re-education and diagnosis. Data show reliability of over 80%.

Exploration of the pelvic floor has been developed through three approaches: transabdominal [93,94], transperineal [95,96], and intracavitary [97,98]. The transabdominal approach [9] allows the probe to be drifted in three spatial axes of about 10º each, without any measuring errors. In this study, respiration, ASLR and ADIM maneuvers were associated.

Additionally, the validity of transabdominal exploration has already been widely demonstrated [95], finding an intra-observer ICC = 0.750–0.943 and an inter-observer ICC = 0.886, with a Cronbach’s alpha of 0.865, both in men and women. In some cases, they were correlated with a gold standard, such as EMG, intra-abdominal pressure control and perineal muscle EMG [95]. High reliability correlations were found (R^2^ = 0.74–0.88) for specific motor control maneuvers.

The use of US was also interesting in order to determine the best position for pelvic floor assessment, comparing it with manometry and digital muscular test in three different positions commonly used at a clinical level (supine, supine with flexed knees, sitting and standing) [99]. The conclusion was that the standing position allows for greater movement of the pelvic floor than supine (*p* = 0.003) and sitting (*p* = 0.001) positions. Another study reaches the same conclusion [100], reinforcing the criteria for clinical applicability.

Regarding the clinical utility of the transducer for this region, a study was presented where two techniques were compared, pelvic floor contraction and the Valsalva maneuver [93], with a resulting concordance level of 85% for pelvic floor contraction and 100% for the Valsalva maneuver. It should be highlighted that the standard error measurements were SEM = 0.91–0.93 for contraction and SEM = 0.87–0.51 for the Valsalva maneuver, and concordance level in the direction of bladder movement during contraction was K = 0.56, *p* < 0.0001. Although both approaches are valid, the transperineal approach is more reliable, whereas the transabdominal approach is faster, therefore more useful. Along this line, the same author later published another study where she focused on the same approaches with functional maneuvers, but assessing the neck and base of the bladder [101]. In that study, she concluded that the transperineal approach offers a higher reliability in obese patients.

The results obtained from the pelvic floor assessment of patients with lumbopelvic pain versus healthy subjects [22] showed that there was less movement in the voluntary contraction of patients versus healthy subjects (*p* = 0.04), with an ICC = 0.87.

Comparison between vaginal palpation, vaginal compression, EMG and US [102] was conducted, resulting in a high correlation between muscle function and pressure in contraction (0.90), a bit lower between these two and EMG (0.52 and 0.60) and movement (0.51 and 0.60). Additionally, it was done for the male pelvic floor [95], with high correlation too (R^2^ = 0.74–0.88). Due to the validity that EMG had always had against US in muscle activation, these results are significant for clinical settings.

Further evidence supports the use of the transducer for transperineal approach, with enough validity to be used at a clinical level. In a study with patients with urinary incontinence [23], intra- and inter-rater reliability was found at rest, squeezing (in contraction) and straining (Valsava maneuver) coincident with others [93] in the pubococcygeus muscle. This also happened for the elevator ani muscle in the same type of patients [103], with statistically significant differences.

Additionally, it is worth mentioning the study performed in patients with vestibulodynia regarding the morphology and muscle function [104], in which morphological differences were found.

One last US assessment technique should be mentioned, the transvaginal approach. In a study where the three approaches described above [105] were compared for bladder thickness assessment, better intra-rater correlation was observed with the transvaginal approach than with the transabdominal and transperineal. Therefore, this approach should be considered the only valid one for the bladder wall, hence becoming of interest for patients with urogynecological disorders [106,107].

Studies about the impact of the transducer in physiotherapy reinforce their use. We observed that the first study published about the use of the transducer in physiotherapy was carried out in South Australia, and more than 600 responses [108] were obtained, out of which only 11.6% used the transducer. Over time, polls on the same topic [109] found a substantial change in the responses obtained. Additionally, in the UK [110], USA [111], New Zealand [112], Spain [113], recently published the results from the international survey [114] confirmed the change of paradigm started by Dr. Young and continued by Dr. Stokes.

Regarding the previous systematic and/or literary reviews published, the following conclusions were made: the US compared with the MRI showed high reliability [26], and that the hyper-echogenic areas were correlated with atrophic areas, demonstrating the validity of this technique for the evaluation of submaximal isometric contractions [25] with good results; validity for the muscle thickness of abdominal and lumbar muscles and CSA of lumbar [24] with high ICC results; low consistency in US versus EMG comparison [27] evaluating the ADIM technique; and the evaluation as a biofeedback tool suggests the need to develop more methodological research and homogeneous studies which can be compared [29], coining the term “RTUS”.

A literary review about pelvic floors in patients with urinary incontinence and/or prolapse [28] concluded there is internal validity in US assessments, but its external validity needs to be assessed.

### Assessment of Techniques. Biofeedback

The evidence founded in this study suggests that it is possible to justify the suitability for enhancing the interventions and the patient experience or reject any hypothesis which are still accepted today. Manual techniques have been evaluated—fascial treatment in abdominal muscles [115], spinal stabilization, manual therapy and pain treatment conducted by a physiotherapist [95]— and, testing the results and checking the increase of sliding and thickness of muscles in fascial treatment, no difference was found between the techniques for the second one.

Regarding exercise-based therapy, there are some conflicts [116,117] due to reporting bias and discrepancies in standards (coinciding with the results of our meta-regression). Pilates demonstrated good results for the increase of TrA muscle contraction [118], and the ADIM maneuver [40,119] would be a useful and specific technique for this muscle. Two studies demonstrated better results in patients educated to do core-control exercises [27] and isometric exercises [120] versus traditional indications, and even better results for those who used pressure units for biofeedback [121]. However, other comparisons revealed no significant differences for muscle thickness, such as Mckenzie versus Motor control therapies [42].

Pelvic floor US was used to check the effect of some interventions, like traditional pelvic floor exercises [122] monitored with US, such as shoulder bridge, abdominal pressure, tiptoes or clam pilates exercises, revealing that the latter exercises were the most effective. The increase of knowledge provided by new strategies such as hypo-pressive exercises [123,124] has made it possible to access new strategies, concluding that there were significant changes (*p* < 0.05), so this therapy is suggested as a therapy of choice. Another study compared three groups, pelvic floor exercises, hypo-pressive exercises, and a control group [124], on the elevator ani muscle in women with prolapse, resulting in no significant differences (*p* < 0.001) between the groups that did exercise.

The invasive procedures in physical therapy have not yet been evaluated with and without US. It must be taken into account that previous studies in sport medicine have demonstrated the high risk of bias when performing invasive procedures that are not echo-guided [125,126].

The diaphragm remains to be correlated in pathological cases. Furthermore, M-mode evaluation has been validated [127] as very useful in avoiding the old fluoroscopy technique [128] in extubating and B-mode technique [129]

## 5. Conclusions

The studies analyzed provide answers to the goals of this review, and most of them offer excellent methodological quality; however, few of them are randomized clinical studies.

The forest plot study in the meta-analysis shows that motor control exercises are effective in the treatment of low back pain, obtaining better results compared to other treatments, because of an increase in muscle thickness and contraction capacity, which can be shown with an US. While is seems very important to consider the timing of application of therapeutic exercise, the meta-regression suggests this is not important because it does not affect the results in the records analyzed.

There is a wide diversity of applications, and this technique has demonstrated a great strength of knowledge in the resting state, functional activities, morphological changes in pathology context, and high validity and reliability in each area (lumbar, abdominal, and pelvic floor).

The transducer has got high reliability versus “gold standards” like MRI and EMG. It has become the first choice for researchers in physiotherapy as result of the huge evidence published, but further specific training is necessary.

The diagnosis for physiotherapy care can be assessed through US, according to world organizations declarations. The transducer provides relevant images on the shape or behavior of tissues after validated tests to help assess a differential diagnosis that determines different types of care and better results.

Thanks to the use of the transducer in physiotherapy, intervention has been measured objectively, providing the best dosimetry in some cases or eligibility in others. Muscle thickness could be related to changes in pain and dysfunction, so its assessment might help to administer and adjust the exercise-based treatment or manual therapy. Applying treatment techniques with a transducer in physiotherapy has been proven to reduce patient training time, because it results in a great biofeedback, achieving better results in less time and maintaining the benefits obtained, although certain studies are controversial.

### Future Lines of Research

The implication of the diaphragm muscle in the lumbopelvic region should be assessed. This muscle has not been analyzed and there are no data about its activation and/or intervention through US. Studies about cost-effectiveness of the clinical use of the transducer in physiotherapy are necessary. Exercise and the use of biofeedback have been proven as the best treatment for lumbar pain, but a deeper knowledge of the effects of exercise-based therapy is necessary in order to determine the right dosage to obtain the best results.

Neither the sequence nor the progression that the patient should follow has been quantified to this day.

In spite of the wide number of studies about US in the lumbopelvic region and about its implication in pain generation, there is no structured protocol for patient assessment at a clinical level.

Further studies are necessary regarding the cost-effectiveness and decision-making confidence regarding the use of the transducer in clinical practice and to improve US application in the clinical practice of physiotherapists. Thus, clinical practice guidelines should be elaborated, in order to systematize patient exploration, and to ensure homogeneity of clinical practice.

## 6. Other Information

### Registration and Protocol

This systematic review was registered in the PROSPERO database (number CRD42017078326).

## Figures and Tables

**Table 1:** Summary of included studies for narrative synthesis (*n* = 24).

Region	Study	Design	Descriptive Statistics	Intervention	Control	Measured Outcomes
**Abdominal**	Teyhen et al. (2005)	RCT	n= 30 Age (y) 30.8 (±10.1) 31.2 (±7.5) Height (cm) 170.7 (±9.5) 169.5 (±7.3) Body mass (kg) 77.9 (±14.1) 77.3 (±8.2)	Biofeedback Trainning (BT)	Traditional Trainnig (TT)	Main outcome: Abdominal muscles thickness
	Chon etal. (2009)	RCT	Experimental (n = 20) Control (n = 20) Age (years) 24 (±1.6) 24 (±1.9) Height (cm) 168 (±8.9) 169 (±7.9) Weight (kg) 61 (±12.0) 59 (±9.1)	ADIM (Abdominal Draw in Maneouvre) + ankle dorsiflexion	ADIM	Main outcome measures: Ultrasonography muscle thickness and electromyography activity of abdominal muscles
	Costa et al. (2009)	RCT	n=35 (22 female) Age (years) 53.3 (11.27) Weight (kg) 69.3 (11.49) Height (m) 1.6 (0.08)	Motor Control Exercise (MCE)	Placebo	To test the automatic recruitment of the abdominal wall muscles by real-time ultrasound imaging
	Bajaj et al. (2010)	Observational	RUSI group (n=11) PBU group(n=11) Age (yrs) MEAN + SD 30.90 + 8.96 32.54 + 6.57 Height (cms) MEAN + SD 163.27 + 9.59 161.30 + 10.41.Weight (kgs) MEAN + SD 59.63 +8.64 58.68 + 9.79 BMI(kg/m2) MEAN + SD 22.5 + 1.4 22.4 + 1.58	RUSI + ADIM	PBU (Pressure Biofeedback Unit) + ADIM	The variables available for analysis were number of days and number of trials for both RUSI and PBU groups
	Vasseljem et al. (2010)	RCT	UGE (n=36) SE (n=36) GE (N=37) Age 40.9 (11.5) 43.4 (10.2) 36 (10.3) BMI 24.9 (3.1) 24.9 (3.1) 24.3 (2.8)	The ultrasound guided exercise (UGE)	Sling Exercise (SE)	1. Muscle Thickness External Oblique, Internal Oblique, Transversus Abdominis (EO, IO, TrA) 2. Pain Numeric Rating Scale (NRS)
	Guthrie et al. (2012)	RCT	n= 51 men (18) Age (y) 23.1 ± 6.0, Height (cm) 173.6 ± 10.5, Mass (kg) 74.7 ± 14.5, BMI (kg/m2) 24.6 ± 2.8	Traditional bridge (TB)	Suspension-exercise bridge (SE)	Main outcome: Abdominal muscles thickness by US
	Ferreira et al. (2014)	RCT	MCE (n = 11) GE (n = 10) SMT (n = 13) Age, years (SD) 47.5 (±17.3) 54.9 (±11.3) 45.4 (±17.7) Weight, kg (SD) 78.7 (±13.0) 70.1 (±12.0) 72.6 (±10.2) Height, cm (SD) 171.0 (±10.8) 160.7 (±6.6) 165.0 (±8.5) Female, n (%) 6 (55) 7 (70) 10 (77)	Motor Control Exercise (MCE)	General Exercise (GE)	TrA was measured using a US. Global impression of recovery. Disability was measured using the Roland Morris disability questionnaire. Pain intensity on a numerical rating scale. Function was measured with a modified patient specific functional scale
	Tajiri et al. (2014)	RCT	Exercise group (n= 9) 52.1 ± 9.5 Height 156.1 ± 6.2 Weight 51.9 ± 5.3 Control group (n= 6) 52.0 ± 7.6 Height 161.0 ± 7.4 Weight 55.7 ± 13.9	TA (Transversus Addominis) + PFM (Pelvic Floor Muscle) co-contration exercise	Control Group (CG)	Authors evaluated the thickness of the TA using ultrasound
	Gisela Rochade et al. (2015)	RCT	Age 31 (5) 30 (8) Weight (kg) 65.2 (9.8) 68.9 (11.7) Height (m) 1.67 (0.07) 1.67 (0.11) BMI (m2/kg) 23.2 (2.0) 24.5 (2.8)	Pilates	Strength	The aim of this study was to compare the effects of Pilates mat exercises and a conventional strength training programme on the activity of TrA and OI. They used ultrasound measures of muscle thickness as a proxy of muscle activity
	Gong et al. (2016)	RCT	Training group (n = 15) 27.35 ± 6.16 164.47 ± 8.32 57.70 ± 8.06 Control group (n = 15) 27.88 ± 6.99 165.00 ± 8.22 59.05 ± 9.96	Running in place	ADIM	Ultrasonography was used to examine the abdominal muscle thicknesses before and after running in place.
	Halliday et al. (2016)	RCT	Age (years) Mckenzie: 48.8 (±12.1) MCE: 48.3 (±14.2) Sex (males); n (%) McKenzie: Males 7 (20.0%) MCE: Males 7 (20.0%)	Mckenzie (MKZ)	Motor Control Exercise (MCE)	1. Muscle Thickness (EO, IO, TrA) 2. Patient Specific Functional Scale 3. Pain (VAS)
	Hoppes et al. (2016)	RCT	n= 34 16 Male, 18 Female Age CG 27 ± 5 MCE 29 ± 5 Weight CG 70.53 ± 15.42 MCE 70.86 ± 10.83 Height CG 1.73 ± 0.11 MCE 1.73 ± 0.12 BMI CG 23.27 ± 2.88 MCE 23.66 ± 2.59	Motor Control Exercise (MCE)	Control Group (CG)	The measures during the pre- and post-intervention assessments included ultrasound imaging of abdominal muscle thickness
	Shamsi et al. (2016)	Q-RCT	Core stability exercise group General exercise group n= 22 n= 21 Male: 7 Male: 6 Female: 15 Female: 15 Age (year) 39.2 ±11.7 Height (cm) 166.4 ±9.1 Weight (kg) 70.1 ±15.1	Motor Control Exercise (MCE)	General Exercise	Using ultrasound imaging, four transabdominal muscle thicknesswere measured before and after the intervention. Disability and pain were measured as secondary outcomes
	Nabavi et al. (2017)	RCT	Stabilization Group Routine Group Mean Standard Age (y) 40.75 ±8.23 34.05 ±10.75 Height (m) 1.68 ±0.08 1.65 ±0.08 Weight (kg) 70.15 ±14.53 72.05 ±10.77 Body mass index (kg/m2) 24.86 ±4.39 26.39 ±3.21	MCE (Motor Control Exercise)+electrotherapy (N=20)	General Exercise + Electrotherapy	Pain intensity, using a visual analog scale, and muscle dimensions of both right and left transverse abdominis and lumbar multifidus muscles, using rehabilitative ultrasonography
	Worth et al. (2007)	RCT	Male 6 (60.0%) 4 (44.4%) Female 4 (40.0%) 5 (55.6%) Age (years) 37.0 ±11.5 33.1 ±13.5 Height (m) 1.74±0.14 1.73±0.12 Weight (kg) 79.0 ± 9.08 73,2 ±14.89	AHE (Abdominal Hollowing Exercise)	AHE + RTUS (Real Time Ultraound)	NPI= Numeric Pain index TCi = Typicai Clinicai Instruction Group TCi + US = Typical Clinical Instruction augmented with Real Time Ultrasound Group
**Lumbar**	Hides et al. (1996)	RCT	Age 30.9 and 30.65 Height 173.3 cm and 170.1 cm Weight 73.53 Kg and 71.05 Kg.	Medical Treatment + Specific localized exercise therapy (T+SET)	Medical Treatment (MT)	Pain, McGill Pain Questionnarie (MPQ), VAS and daily pain diaries. The Roland Morris Disabiliy Index. Range of motion, and size of the multifidus cross-sectional area (CSA)
	Van et al. (2005)	RCT	Group 1 (knowledge of results [KR] alone) contained 10 females and 3 males (mean ± SD, 19.1 ± 2.1 years) and group 2 (KR plus visual feedback) contained 9 females and 3 males (mean ± SD, 19.9 ± 2.2 years).	Clinical instructions for multifidus muscle contraction + Provision of visual biofeedback using real-time ultrasound imaging	Clinical instructions for multifidus muscle contraction	Multifidus muscle thickness
	Akbari et al. (2008)	RCT	MCE (n = 25) GE(n = 24). Age 39.6 ± 3.5b 40 ± 3.6. Height (cm) 171.2 ± 2.7 172.08 ± 2.2 0.2 Weight (kg) 73.7 ± 3.1 74.6 ± 2.4 0.26 BMI (kg/m2) 25.2 ± 1.7 25.21 ± 1.02	Motor Control Exercise (MCE)	General Exercise (GE)	1. Muscle Thickness Transversus Abdominis and Lumbar Multifidus (TA and LM) 2. Activity limitation (AL) was assessed using Back Performance Scale (BPS). 3. Pain measurement Visual Analog Scale (VAS)
	Hebert et al. (2015)	RCT	MT (n=20) MT+SET (n=21) Age 31 ± 7.9 - 30.9 ± 6.5 Height 173.3 - 170.1 Weight 73.53 - 71.05	Specific Trunk Exercises (MCE)	General Trunk Exercise (GE)	1. Pain: McGill Pain Questionnarie (MPQ) and Visual Analogue Scale (VAS) 2. Disability: Roland Morris Disability Index (RMDI) 3. Range of Motion: Inclinometry 4. Habitual activity levels 5. Lumbar multifidus Muscle CSA (LM)
	Berglund et al. (2017)	RCT	LMC n=33 Age 43.3 (10.3) BMI 25.0 (3.0) HLL n=32 Age 42.3 (9.8) BMI 25.4 (3.8)	Low Load Motor Control Exercises (LMC)	High-Load lifting (HLL) Exercise	Pain (VAS), Multifidus mucles thickness
**Pelvic Floor**	Stuge et al. (2006)	RCT	Weight (kg) 69.5 (11.7) 67.3 (13.6) Height (cm) 169.6 (3.6) 164.5 (5.4) Body mass index 24.1 (3.3) 25.0 (5.4) Age of youngest child (months) 29.5 (2.9) 29.6 (3.6)	Volunteers with PGP (Pelvic Girdle Pain) + ASRL (Active Straigh Raise Leg)	Volunteers without PGP + ASRL	Abdominal muscles thicknes by ultrasound, pelvic floor muscles strength by pressure transducer, ability to perform ASLR test, Pain (VAS)
	Bernardes et al. (2012)	RCT	Age (years) 51.9 (± 7.4) 56.7 (± 10.7) 58.7 (± 10.4) Body mass index (BMI, kg/m2) 29.9 (± 3.5) 28.8 (± 3.9) 29.7 (± 2.7)	**Pelvic floor muscle training** group (GI)	Hypopressive exercise group (GII)	Ano rectal muscle CSA, Length of urethra adn bladder neck by transperineal ultrasound. Pelvic organ prolapse (POP) classification
	McLean et al. (2013)	RCT	Control group 54.0 ±8.4 years, treatment group 49.5 ±8.2 years, body mass index (control group 28.6 ±11.3 kg/m2, treatment group 27.0 ±3.8 kg/m2)	12 weekly sessions they learned **contract their pelvic floor muscles** (PFMs) and a home exercise program	No treament.	Baldder volume by trans-abdominal US, transperineal ultrasound for urethra morphology, Incontinence Impact Questionnaire (IIQ-7) and the Urogenital Distress Inventory (UDI-6)
	Johannessen et al. (2016)	RCT	Intervention group (n = 54) Control group (n = 55) Age (years), mean (SD) [range] 29.7 (4.3) [20,21,22,23,24,25,26,27,28,29,30,31,32,33,34,35,36,37,38] 30.6 (3.8) [23,24,25,26,27,28,29,30,31,32,33,34,35,36,37,38,39,40] Inclusion (days postpartum), mean (SD) 389 (122) 375 (141) Ethnicity: Norwegian 42 (77.8) 51 (92.4) European 8 (14.8) 3 (5.5) Asian 4 (7.4) 1 (1.8)	Individual physiotherapya of pelvic floor muscle exercises PFME (Pelvic Floor Muscle Exercise)	Written information of (PFME)	St. Mark’s score for Anal Incontinence, manometry measures of anal sphincter length and strength, endoanal ultrasound (EAUS) defect score and voluntary pelvic floor muscle contraction
**Legend: RCT: Randomized Clinical Trial, Q-RCT: Quasi Randomized Clinical Trial. EO (External Oblique), IO (Internal Oblique), TrA (Transversus Abdominus), US (Ultrasound), PBU (Pressure Biofeedback Unit), MCE (Motor Control Exercise).**						

**Table 2:** Summary of included studies for quantitative synthesis (n = 6).

Region	Author	Design	Title	Intervention	Control	Descriptive Statistics	Measured Outcomes
**LUMBAR**	Akbari et al. (2008)	RCT	The effect of motor control exercise versus general exercise on lumbar local stabilizing muscles thickness: Randomized controlled trial of patients with chronic low back pain	Motor Control Exercise (MCE)	General Exercise (GE)	MCE (n = 25) GE(n = 24). Age 39.6 ± 3.5b 40 ± 3.6. Height (cm) 171.2 ± 2.7 172.08 ± 2.2 0.2 Weight (kg) 73.7 ± 3.1 74.6 ± 2.4 0.26 BMI (kg/m2) 25.2 ± 1.7 25.21 ± 1.02	1. Muscle Thickness (TA and LM) 2. Activity limitation (AL) was assessed using Back Performance Scale (BPS). 3. Pain measurement Visual Analog Scale (VAS)
	Berglund et al. (2017)	RCT	Effects of low-load motor control exercises and a high-load lifting exercise on lumbar multifidus thickness	Low Load Motor Control Exercises (LMC)	High-Load lifting (HLL) Exercise	LMC (n=33) Age: 43.3 (10.3) BMI: 25.0 (3.0) HLL (n=32) Age: 42.3 (9.8) BMI: 25.4 (3.8)	1. VAS (Visual Analogue Scale) 2. Muscle Thickness
**ABDOMINAL**	Ferreira et al. (2014)	RCT	Changes in recruitment of transversus abdominis correlate with disability in people with chronic low back pain	Motor Control Exercise (MCE)	General Exercise	MCE (n = 11) - GE (n = 10) - SMT (n = 13) Age, years (SD) 47.5 (17.3) 54.9 (11.3) 45.4 (17.7) Weight, kg (SD) 78.7 (13.0) 70.1 (12.0) 72.6 (10.2) Height, cm (SD) 171.0 (10.8) 160.7 (6.6) 165.0 (8.5) Female, n (%) 6 (55) 7 (70) 10 (77)	Global impression of recovery was measured on an 11-point scale. 1.Disability was measured using the 24-item version of the Roland Morris disability questionnaire. 2.Average pain intensity over the past week was measured on a numerical rating scale. 3.Function was measured with a modified patientspecific functional scale
	Halliday et al. (2016)	RCT	A Randomized Controlled Trial comparing the Mckenzie method to motor control exercises in people with chronic low back pain and a directional preference.	Motor Control Exercise (MCE)	Mckenzie (MKZ)	Age (years) Mckenzie: 48.8 (12.1) MCE: 48.3 (14.2) Sex (males); n (%) McKenzie: Males 7 (20.0%) MCE: Males 7 (20.0%)	1. Muscle Thickness (EO, IO, TrA) 2. Patient Specific Functional Scale 3. Pain (VAS)
	Shamsi et al. (2016)	Q-RCT	The effect of core stability and general exercise on abdominal muscle thickness in non-specific chronic low back pain using ultrasound imaging	Motor Control Exercise (MCE)	General Exercise	Core stability exercise group General exercise group N = 22 N = 21 Male: 7 Male: 6 Female: 15 Female: 15 Age (year) 39.2 11.7 Height (cm) 166.4 9.1 Weight (kg) 70.1 15.1	Using ultrasound imaging, four transabdominal muscle thickness were measured before and after the intervention. Disability and pain were measured as secondary outcomes
	Nabavi et al. (2017)	RCT	The effect of 2 different exercise programs on pain intensity and muscle dimensions in patients with chronic low back pain: A randomized controlled trial	MCE (Motor Control Exercise) +electrotherapy (N=20)	General Exercise + Electrotherapy (N=21)	Stabilization Group Routine Group Mean Standard Age (y) 40.75 8.23 34.05 10.75 Height (m) 1.68 0.08 1.65 0/08 Weight (kg) 70.15 14.53 72.05 10.77 Body mass index (kg/m2) 24.86 ±4.39 26.39 ±3.21	Pain intensity, using a visual analog scale, and muscle dimensions of both right and left transverse abdominis and lumbar multifidus muscles, using rehabilitativeultrasonography
**Legend: RCT: Randomized Clinical Trial, Q-RCT: Quasi Randomized Clinical Trial.**							

## Data Availability

The datasets used and/or analyzed in the current study or any query regarding to the research process are available from the corresponding author.

## Supplementary Materials

The following are available online at https://www.mdpi.com/article/10.3390/jcm10235699/s1, File S1: Search_strategy, File S2: Risk of Bias Assessment, File S3: Risk of Bias Tables, File S4: Complete annotated Forest plot-graphs and tables.
