# Supplementary material for: The Role of Rehabilitative Ultrasound Imaging Technique in the Lumbopelvic Region as a Diagnosis and Treatment Tool in Physiotherapy: Systematic Review, Meta-Analysis and Meta-Regression
Source: J Clin Med. 2021 Dec 3;10(23):5699. doi: 10.3390/jcm10235699 (PMC8658262; doi:10.3390/jcm10235699)
Supplement: Supplementary file 1 [file jcm-10-05699-s001.zip › jcm-1398772-supplementary.pdf]

Supplementary File S1: Search strategy

Fernández-Carnero S, Martin-Saborido C, Achalandabaso Ochoa-Ruiz de Mendoza A, Ferragut-Garcias A, Cuenca-Zaldivar JN, Leal-Quiñones A, Calvo-Lobo C, Gallego-Izquierdo T

The role of Rehabilitative Ultrasound Imaging Technique in lumbopelvic region as a diagnosis and treatment tool in Physiotherapy. Systematic Review, meta-analisis and meta-regression.

Databases: ScienceDirect, Medline, SportDiscus, CINHAL, Cochrane Database of Sistematic Review, SciELO, EMBASE

A search strategy was created in consultation with a medical librarian by combining MeSH and key terms in a manner that resulted in 3 important concepts from our research question: ultrasound, specific muscles, and the trunk region. The search was no idiom limitation. Neither limittion about the publication types (academic publications, books, journals, conferences, thesis…). Three search lines were made, one by muscular region in core.

1. **Key words: Lumbar region.** ('rehabilitative ultrasound imaging' OR 'ultrasound imaging' OR echography OR ultrasonography OR 'real time ultrasound imaging') AND ('lumbar spine' OR 'lumbar region' OR 'lumbar multifidus' OR 'low back') AND [1994-2017]/py

2. **Key words: Abdominal wall.** ('rehabilitative ultrasound imaging' OR 'ultrasound imaging' OR echography OR ultrasonography OR 'real time ultrasound imaging') AND ('abdominal wall' OR 'abdominal wall musculature') AND [1994-2017]/py

3. **Key words: Pelvic Floor.** ('rehabilitative ultrasound imaging' OR 'ultrasound imaging' OR echography OR ultrasonography OR 'real time ultrasound imaging') AND ('pelvic floor' OR 'endopelvic fascia' OR 'bladder base') AND [1994-2017]/py

Supplementary File S2: Risk of Bias Assessment

Fernández-Carnero S, Martin-Saborido C, Achalandabaso Ochoa-Ruiz de Mendoza A, Ferragut-Garcias A, Cuenca-Zaldivar JN, Leal-Quiñones A, Calvo-Lobo C, Gallego-Izquierdo T

The role of Rehabilitative Ultrasound Imaging Technique in lumbopelvic region as a diagnosis and treatment tool in Physiotherapy. Systematic Review, meta-analisis and meta-regression.

The Risk of Bias REVMAN´s tool

**Bias**	**Authors' judgement**	**Support for judgement**
Random sequence generation (selection bias)	Low risk<br>Unclear risk<br>High risk	
Allocation concealment (selection bias)	Low risk<br>Unclear risk<br>High risk	
Blinding of participants and personnel (performance bias)	Low risk<br>Unclear risk<br>High risk	
Blinding of outcome assessment (detection bias)	Low risk<br>Unclear risk<br>High risk	
Incomplete outcome data (attrition bias)	Low risk<br>Unclear risk<br>High risk	
Selective reporting (reporting bias)	Low risk<br>Unclear risk<br>High risk	
Other bias	Low risk<br>Unclear risk<br>High risk	

Risk of bias table

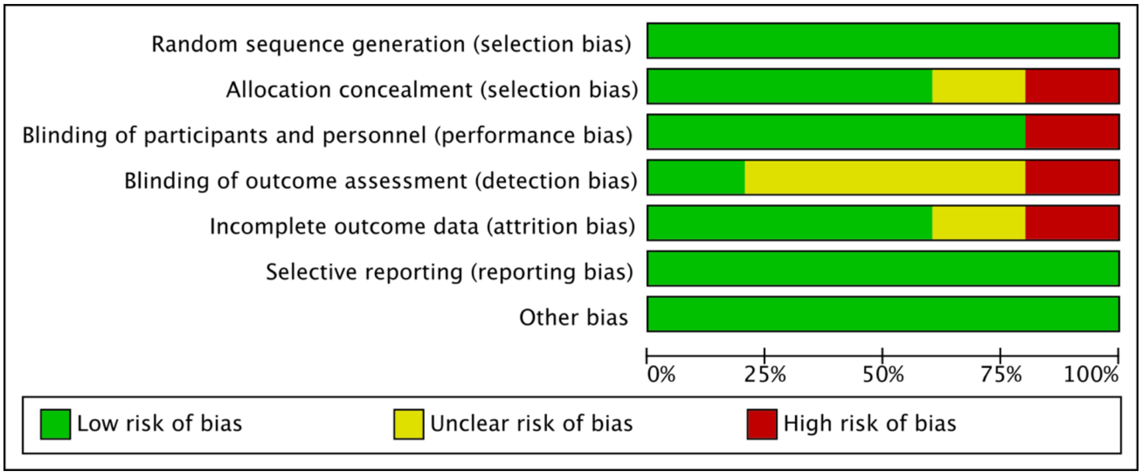

Risk of bias graph.

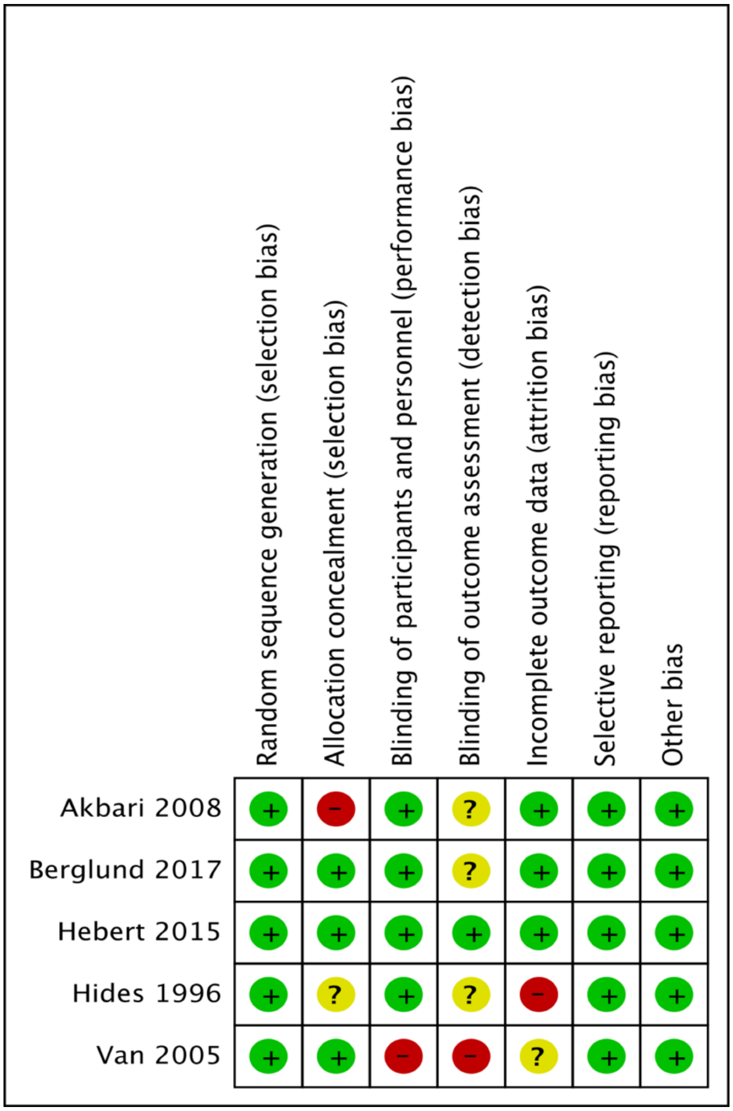

Risk of bias summary (lumbar region example)

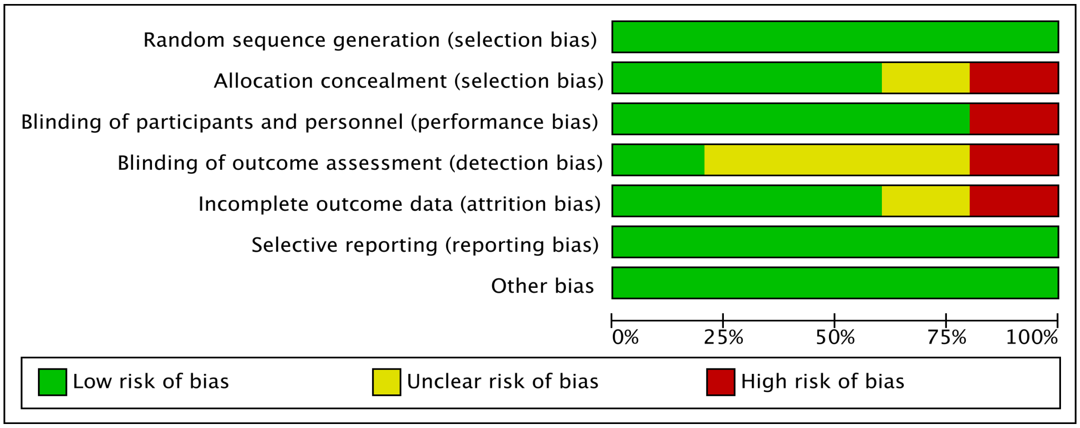

Graphic S1 Risk of bias graph: review authors' judgements about each risk of bias item presented as percentages across all included studies for lumbar.

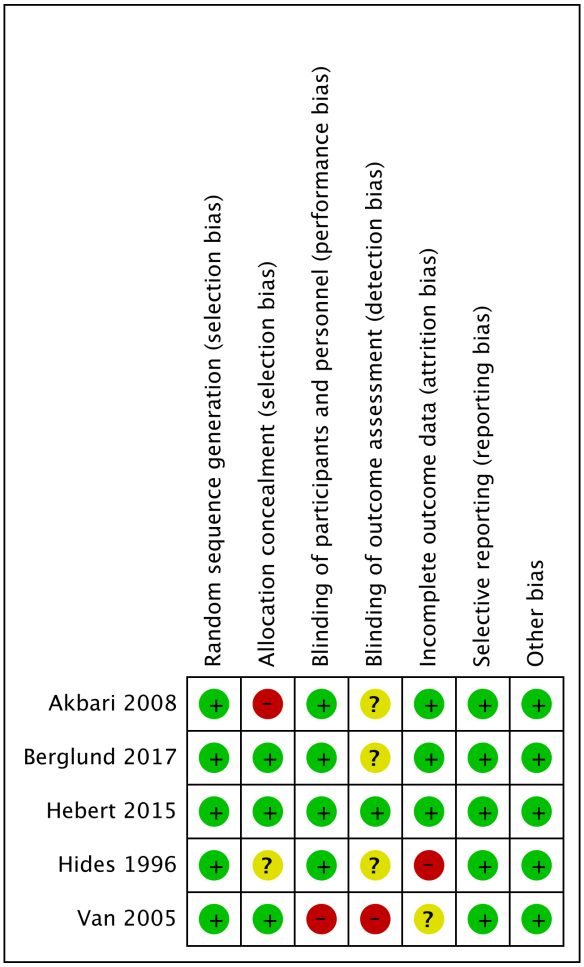

Graphic S2 Risk of bias summary: review authors' judgements about each risk of bias item for each included study for lumbar.

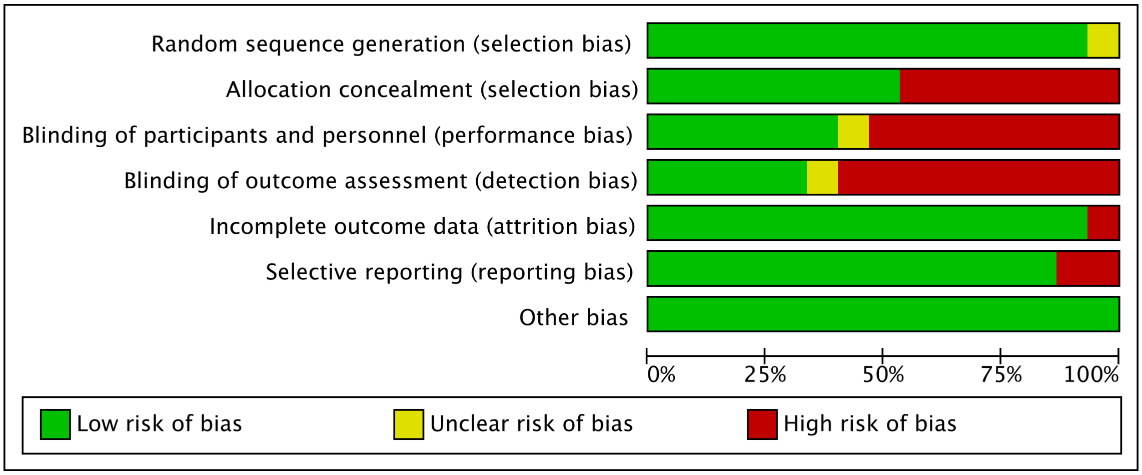

Graphic S3 Risk of bias graph: review authors' judgements about each risk of bias item presented as percentages across all included studies for abdomen.

	Random sequence generation (selection bias)	Allocation concealment (selection bias)	Blinding of participants and personnel (performance bias)	Blinding of outcome assessment (detection bias)	Incomplete outcome data (attrition bias)	Selective reporting (reporting bias)	Other bias
Bajaj 2010	+	−	−	−	+	+	+
Chon 2010	+	+	+	?	+	+	+
Costa 2009	+	+	+	−	−	−	+
Ferreira 2014	+	+	+	+	+	+	+
Gisela Rochade 2015	+	−	−	−	+	+	+
Gong 2016	+	−	−	−	+	+	+
Guthrie 2012	+	+	+	+	+	+	+
Halliday 2016	+	+	?	+	+	+	+
Hoppes 2016	+	+	−	−	+	+	+
Nabavi 2017	+	−	−	+	+	−	+
Shamsi 2016	?	−	−	−	+	+	+
Tajiri 2014	+	−	−	−	+	+	+
Teyhen 2005	+	+	+	+	+	+	+
Vasseljen 2010	+	+	+	−	+	+	+
Worth 2007	+	−	−	−	+	+	+

Graphic S4 Risk of bias summary: review authors' judgements about each risk of bias item for each included study for abdomen.

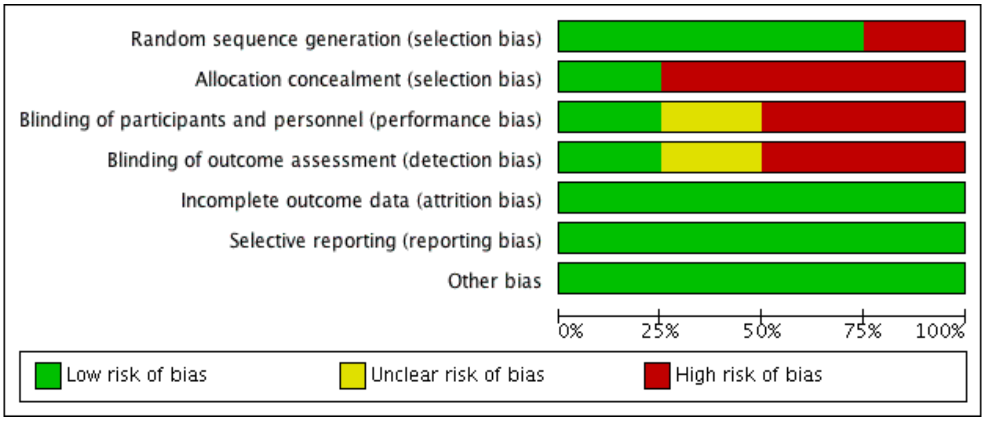

Graphic S5 Risk of bias graph: review authors' judgements about each risk of bias item presented as percentages across all included studies for pelvic floor.

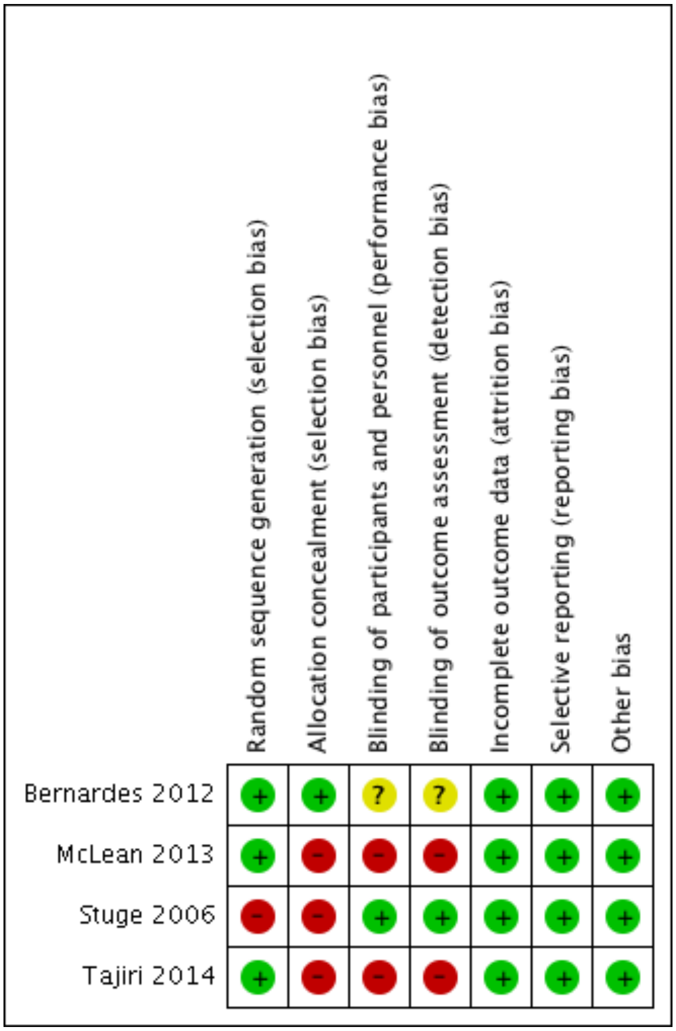

Graphic S6 Risk of bias summary: review authors' judgements about each risk of bias item for each included study for pelvic floor.

Higgins JPT, Green S (editors). *Cochrane Handbook for Systematic Reviews of Interventions* Version 5.1.0 [updated March 2011]. The Cochrane Collaboration, 2011. Available from www.handbook.cochrane.org.

Supplementary File S3: Risk of Bias Tables

Fernández-Carnero S, Martin-Saborido C, Achalandabaso Ochoa-Ruiz de Mendoza A, Ferragut-Garcias A, Cuenca-Zaldívar JN, Leal-Quiñones A, Calvo-Lobo C, Gallego-Izquierdo T

The role of Rehabilitative Ultrasound Imaging Technique in lumbopelvic region as a diagnosis and treatment tool in Physiotherapy. Systematic Review, meta-analisis and meta-regression.

Supplementary File 3: The Characteristics of the studies & Risk of Bias Tables with evidence.

The Characteristics of the studies & Risk of Bias Tables with evidence

LUMBAR

Akbari 2008

**Methods**	*"This was a double-blind, randomized controlled trial with patients randomly assigned to 1 of 2 treatments group. The first group treated with the motor control exercise and the other with general exercise. The physical therapist who administered the exercise programs could not be masked to group allocation. The radiologist who measured muscles thickness, the researcher who evaluated the pain and activity limitation and analyzed the data and participants were blinded to group assignment. The University Ethics Committee approved the protocol of the study, and all patients gave their written voluntary informed consent before participation"*
**Participants**	*"Forty-nine patients with chronic LBP were randomly assigned to either a motor control (n = 25) or a general exercises group (n = 24).<br>Eligible participants were screened for contraindications to exercise using the Physical Activity Readiness Questionnaire (PARQ). If a subject gave a positive response to items 1, 2, 3, 4, 6 or 7, for medical review and excluding any contraindication to exercise as listed in the ACSM guidelines referred to physician"*
**Interventions**	*"- Motor control exercise: The motor control exercise program is based on the treatment approach reported by O'Sullivan et al (P.B. O'Sullivan, L.T. Twomey and G.T. Allison, Evaluation of specific stabilizing exercise in the treatment of chronic low back pain with radiologic diagnosis of spondylolysis or spondylolisthesis, Spine 22(24) (1997), 2959–2967.)<br>- General exercise: This exercise activates paravertebral and abdominal muscles. Because this exercise impose extra loading on the spinal tissues, the general exercise was selected on the basis of maximizing the contraction benefit/spinal loading ratio, according to recommendations provided from recent experimental studies. (S.M. McGill, Low back exercises: evidence for improving exercise regimens, Phys Ther 78(7) (1998), 754–765.)"*
**Outcomes**	*"- Muscle thickness measurement: TA and LM thickness (mm) were assessed using a 7.5 MHz B-mode transducer ultrasound (Sonoline Adara; Siemens Medical System, Inc; Issaquah,WA, USA)<br>- Activity limitation assessment: Activity limitation was assessed using Back Perfor- mance Scale (BPS). BPS consists of 5 tests (Sock Test, Pick-up Test, Roll-up Test, Finger tip-to-Floor Test, and Lift Test), all requires sagittal-plane mobility.<br>- Pain measurement: The Visual Analog Scale (VAS) was used to assess each patient's pain perception. It is a responsive pain scale that yields reliable and valid data. VAS rated on an intensity scale from 0 to 100 mm, with higher scores representing higher levels of pain"*
**Notes**	

Risk of bias table

Bias	Authors' judgement	Support for judgement
Random sequence generation (selection bias)	Low risk	*"Patients were randomized through a physical therapist generated random number sequence".*
Allocation concealment (selection bias)	High risk	*"The physical therapist who administered the exercise programs could not be masked to group allocation"*
Blinding of participants and personnel (performance bias)	Low risk	*"This was a double-blind, randomized controlled trial with patients randomly assigned to 1 of 2 treatments group........The radiologist who measured muscles thickness, the researcher who evaluated the pain and activity limitation and analyzed the data and participants were blinded to group assignment"*

Blinding of outcome assessment (detection bias)	Unclear risk	*Nothing*
Incomplete outcome data (attrition bias)	Low risk	*Some*
Selective reporting (reporting bias)	Low risk	*"There were no pre-treatment differences between two groups in any of these measures (P >0.05). Figure 1 presents the recruitment strategy and experimental plan"*
Other bias	Low risk	*Nothing*

Berglund 2017

**Methods**	*"To compare the effects of low-load motor control (LMC) exercises and a high-load lifting (HLL) exercise, on lumbar multifidus (LM) thickness on either side of the spine and whether the effects were affected by intensity or change in pain intensity. Here, we investigated the effects on percentage change [(follow-upbaseline/baseline) X 100] in thickness of the LM muscle at the fifth lumbar vertebra at the small and large sides. The study protocol was approved by The Regional Ethical Review Board in Umea (No. 09–200M)"*
**Participants**	*"Sixty-five participants diagnosed with nociceptive mechanical LBP were included and randomized into LMC exercises or a HLL exercise, the deadlift"*
**Interventions**	*"- Low-load motor control (LMC) exercises"*<br>*"- High-load lifting (HLL) exercise"*
**Outcomes**	*"- Pain intensity during the last 7 days (100mm visual analogue scale [VAS 7 days])23 was measured at baseline and at follow-up"*<br>*"- Ultrasound imaging of the thickness of LM muscles at both sides of the fifth lumbar vertebra was conducted by a PT certified in ultrasound imaging"*
**Notes**	*"This study is part of a larger data collection evaluating the effects of LMC exercises and an HLL exercise (NCT01061632). (Aasa B, Berglund L, Michaelson P, et al. Individualized low-load motor control exercises and education versus a high-load lifting exercise and education to improve activity, pain intensity, and physical performance in patients with low back pain: a randomized controlled trial. J Orthop Sports Phys Ther 2015; 45:77–85.)"*

Risk of bias table

Bias	Authors' judgement	Support for judgement
Random sequence generation (selection bias)	Low risk	*"Detailed description of the selection process has been presented earlier.(10.2519/jospt.2015.5021). After the participants gave their consent, the randomization procedure was performed. The randomization was performed by a person who had not been in contact with any of the participants.*<br>*First, the participants were assigned a number in sequence of their enrollment in the study"*
Allocation concealment (selection bias)	Low risk	*"A second investigator thereafter contacted each participant giving times for first appointment. The physical therapist (PT) performing the RUSI measurements, after the intervention period, was blinded to baseline data, but not group allocation"*
Blinding of participants and personnel (performance bias)	Low risk	*Some.*
Blinding of outcome assessment (detection bias)	Unclear risk	*Nothing.*
Incomplete outcome data (attrition bias)	Low risk	*"There was a significant (P<0.001) difference in thickness of the LM muscle between the small and large side for both men and women. This asymmetry ([thickness on large side - thickness on small side]/thickness on large side100)10 was*

		9.3% for men and 8.8% for women"
Selective reporting (reporting bias)	Low risk	*"There were no significant differences between the LMC and HLL groups for baseline values (Table 1). The values for LM thickness and percentage change are described in Table 2. There were no significant differences between intervention groups for the baseline values of the small or large side of the LM muscles. Men had a significantly (P¼0.03) greater LM thickness on the large side and a near significantly (P¼0.06) greater LM thickness on the small side at baseline compared with women"*
Other bias	Low risk	*Nothing.*

Hebert 2015

**Methods**	*"Allocation: randomised using a number generator.<br>Duration: 6 months follow up.<br>Setting: Patients from academic and private neurological and orthopaedic spine surgery practices in Salt Lake City, Utah, USA"*
**Participants**	*"Diagnosis: Post-discectomy surgery.<br>N= 61<br>Age: average age<br>Sex: Male & Female.<br>Inclusion: Age 18–60 years, presurgical radiographic confirmation of lumbar disc herniation<br>through MRI or CT and scheduled to undergo single-level lumbar discectomy<br>Exclusion: Prior lumbar spine surgery, surgery at more than one level, a surgical procedure other than discectomy (eg, fusion) or perioperative complications representing a contraindication to exercise"*
**Interventions**	*"* Group 1: General trunk exercise protocol (GEN) N=32. This protocol comprised three components: (1) aerobic exercise, (2) range of motion exercise and (3) strengthening exercise.<br>* Group 2: Specific trunk exercise protocol (SPEC) N= 29. The SPEC included all components of the GEN. In addition, participants performed specific trunk muscle exercises similar to protocols used to treat patients with non-specific, non-surgical low back pain. This approach also included similar contractions of the transversus abdominis (TrA) using the abdominal drawing-in manoeuvre. Once these skills were acquired and confirmed by the physical therapist through palpation and/or ultrasound imaging, participants were instructed to perform isometric TrA and LM cocontractions. During the supervised exercise sessions, tactile and visual feedback through palpation and real-time ultrasound<br>imaging were used to enhance skill acquisition and the treating<br>physical therapists used this information to ensure appropriate<br>technique"*
**Outcomes**	*"-Low back pain-related disability: Oswestry Disability Questionnaire (OSW).<br>-Low back and lower extremity pain: Numeric Pain Rating Scale .30–32 Global rating of change (GRC) was assessed with a 15-point Likert-type scale ranging from −7 ("a very great<br>deal worse") to 0 ("about the same") to +7 ("a very great deal<br>better").<br>-Sciatica frequency and bothersomeness were estimated using the Sciatica Frequency and Sciatica Bothersomeness indices resulting in possible scores of 0–25.34<br>-Muscle function was assessed using brightness-mode, real time<br>ultrasound images of LM thickness"*
**Notes**	

Risk of bias table

Bias	Authors' judgement	Support for judgement
Random sequence generation (selection bias)	Low risk	*"A random number generator was used to create a permutedblock randomisation list with variable*

		block sizes of 4–6"
Allocation concealment (selection bias)	Low risk	*"Sequentially numbered, opaque envelopes containing the participant's group assignment were prepared by research staff not affiliated with this trial"*
Blinding of participants and personnel (performance bias)	Low risk	*"The envelope was opened after the 2-week postoperative assessment by the treating physical therapist. Group assignments were concealed from participants and outcome assessors"*
Blinding of outcome assessment (detection bias)	Low risk	*"The envelope was opened after the 2-week postoperative assessment by the treating physical therapist. Group assignments were concealed from participants and outcome assessors"*
Incomplete outcome data (attrition bias)	Low risk	*"There were significant main effects of time (p<0.01) indicating improvements from baseline in disability, pain, sciatica frequency, sciatica bothersomeness and LM function (table 3 and figure 2)"*
Selective reporting (reporting bias)	Low risk	*"The results of the intention-to-treat analyses revealed no time by group interactions. There were no statistically significant or clinically important between-group differences in disability, pain, global change, sciatica frequency, sciatica bothersomeness or LM muscle function at 10 weeks or 6 months (table 3 and figure 2)"*
Other bias	Low risk	*Nothing.*

Hides 1996

**Methods**	*"Clinical study was conducted with acute, first-episode, unilateral low back pain and unilateral, segmental inhibition of the multifidus muscle. Patients were allocated randomly to a control or treatment group"*
**Participants**	*"N= 39 patients*<br>*Age: 18-45 years*<br>*Sex: Male and Female.*<br>*Inclusion: Experiencing their first episode of unilateral, mechanical LBP for less than 3 weeks. Pain located between T12 and gluteal fold.*<br>*Exclusion: Previus history of LBP or injury, previus lumbar surgery, spinal abnormalities indicated on radiographs, neuromuscular or joint desease, reflex and/or motor signs of nerve root compression or cauda equina compression, evidence of systemic disease, carcinoma or organ disease, pregnancy and any sports of fitness training involgin the low bac muscles done in the past 3 months"*
**Interventions**	*"Patients in group 1 received medical treatment only. Patients in group 2 received medical treatment and specific, localized, exercise therapy"*
**Outcomes**	*"Outcome measures for both groups included 4 weekly assessments of pain, disability, range of motion, habitual activity levels and size of the multifidus cross-sectional area. Independent examiners were blinded to group allocation. Patients were reassessed at a 10-week follow-up examination"*
**Notes**	

Risk of bias table

Bias	Authors' judgement	Support for judgement
Random sequence generation (selection bias)	Low risk	*"Random assignment to the control (group1, medical management) or the treatment group*

		(grouep2, medical agement and specific, exercise therapy) was achieved by selecting the group number (one or two) from sealed, shuffled envelopes"
Allocation concealment (selection bias)	Unclear risk	*Nothing.*
Blinding of participants and personnel (performance bias)	Low risk	*"Assessment were performed by two independent examiners who were blinded to group allocation and patient presentation"*
Blinding of outcome assessment (detection bias)	Unclear risk	*Nothing.*
Incomplete outcome data (attrition bias)	High risk	*"The data from these patients have not been included in the analyses presented"*
Selective reporting (reporting bias)	Low risk	*"One patient missed the 10-week follow-up examination because of the illness of a family member. Teh frop-out rate after 10 weeks, therefore, was 2.4%"*
Other bias	Low risk	*Nothing.*

Van 2005

**Methods**	*"Healthy subjects were randomly divided into groups that received different forms of biofeedback. To determine if the provision of visual biofeedback using real-time ultrasound imaging enhances the ability to activate the multifidus muscle"*
**Participants**	*"A total of 25 healthy normal adult volunteers aged 18 to 25 years were studied. Exclusion criteria included current LBP, history of LBP, previous lumbar injury or surgery, known neuromuscular or joint disease, significant spinal abnormality (eg, scoliosis), prior experience with biofeedback using ultrasound imaging, prior training in cognitive activation of the multifidus muscle, and any sports or fitness training (greater than 3 times per week) involving the low back muscles performed within the past 3 months"*
**Interventions**	*"All subjects received clinical instruction on how to activate the multifidus muscle isometrically prior to testing and verbal feedback regarding the amount of multifidus contraction, which occurred during 10 repetitions (acquisition phase). In addition, 1 group received visual biofeedback (watched the multifidus muscle contract) using real-time ultrasound imaging. All subjects were reassessed a week later (retention phase)"*
**Outcomes**	*"Ultrasound measure of the multifidus muscles thickness increase as a percentage"*
**Notes**	

Risk of bias table

Bias	Authors' judgement	Support for judgement
Random sequence generation (selection bias)	Low risk	*"Both genders were included with subjects randomly allocated to 1 of 2 groups"*
Allocation concealment (selection bias)	Low risk	*"...by selection of a sealed envelope containing either number 1 or 2"*
Blinding of participants and personnel (performance bias)	High risk	*Can´t be blinded.*
Blinding of outcome assessment (detection bias)	High risk	*Not detalied*
Incomplete outcome data (attrition bias)	Unclear risk	*Participants data not appears*
Selective reporting (reporting bias)	Low risk	*Detailed in Figure 4*
Other bias	Low risk	*Not others*

ABDOMINAL

Bajaj 2010

**Methods**	*"Twenty two patients with chronic low back pain having transverse abdominis muscle weakness were randomized in two groups.... Number of trials and number of days required for learning the correct maneuver were noted as outcome measures for the two groups. To evaluate the patient's learning a retention test was conducted after 2 days"*
**Participants**	*"Twenty two patients with a history of low back pain for more than 3 months duration and with TrA dysfunction participated in the study"*
**Interventions**	*"...for feedback training, one with real-time ultrasound imaging and second with pressure biofeedback training"*
**Outcomes**	*"The variables available for analysis were number of days and number of trials for both RUSI and PBU groups"*
**Notes**	

Risk of bias table

Bias	Authors' judgement	Support for judgement
Random sequence generation (selection bias)	Low risk	*".....Then the patients were randomized into two groups: RUSI Group and PBU group"*
Allocation concealment (selection bias)	High risk	*Not detailed*
Blinding of participants and personnel (performance bias)	High risk	*Not detailed*
Blinding of outcome assessment (detection bias)	High risk	*Not detailed*
Incomplete outcome data (attrition bias)	Low risk	*"The variables available for analysis were number of days and number of trials for both RUSI and PBU groups. The data was managed on an excel spreadsheet and was analyzed using a SPSS software. Descriptive statistics (mean, standard deviation) were computed for each studied variable. For between the group comparison: Unpaired ttest was used for the statistical analysis to compare the effect of dependent variable on independent variable in between the two groups. The level of the significance was fixed at p ≤ 0.01 for the study analysis"*
Selective reporting (reporting bias)	Low risk	*There are comparations reported between groups and days for the variables stablished.*
Other bias	Low risk	*Nothing*

Chon 2010

**Methods**	*"A preliminary, randomised, controlled study"*
**Participants**	*"Forty healthy adults (18 males, 22 females) were allocated at random to the experimental group [mean age (SD) 24 (1.6) years, n = 20] or the control group [mean age (SD) 24 (1.9) years, n = 20]"*
**Interventions**	*"The experimental group performed the abdominal draw-in manoeuvre in combination with ankle dorsiflexion, and the control group performed the abdominal draw-in*

	manoeuvre alone, five times a day"
**Outcomes**	*"Ultrasonography and electromyography"*
**Notes**	

Risk of bias table

Bias	Authors' judgement	Support for judgement
Random sequence generation (selection bias)	Low risk	*"The participants were allocated at random into the experimental group or the control group"*
Allocation concealment (selection bias)	Low risk	*"The investigators responsible for assessing the outcomes were unaware of an individual's group assignment"*
Blinding of participants and personnel (performance bias)	Low risk	*"Random allocation was implemented using the conventional randomisation directory method in which a random number table was used to produce one code card for each participant, who then picked a card to receive his or her group assignment"*
Blinding of outcome assessment (detection bias)	Unclear risk	*Not data.*
Incomplete outcome data (attrition bias)	Low risk	*"A significant difference was found in the thickness of the transverse abdominal muscle between the groups [mean difference 0.24 cm, 95% confidence interval (CI) 0.08 to 0.40, P =0.005. On electromyography, a significant difference was demonstrated in the amplitude of the transverse abdominal muscle contraction between the two techniques in the experimental group (mean difference 68.76mV, 95% CI 53.16 to 84.36, P =0.000"*
Selective reporting (reporting bias)	Low risk	*"The intra-class correlation coefficient (ICC2,1) showed excellent test–retest reliability of ultrasound measurement of the abdominal muscles: 0.96 (95% CI 0.85 to 0.99) for the transverse abdominal muscle, 0.87 (95% CI 0.62 to 0.98) for the internal oblique*

		muscle and 0.77 (95% CI 0.44 to 0.96) for the external oblique muscle"
Other bias	Low risk	*Nothing.*

Costa 2009

**Methods**	*"This study was nested within an existing randomised, blinded, placebo-controlled trial that compared the efficacy of motor control exercise (MCE) versus placebo in patients with chronic non-specific low back pain (Maher CG, Latimer J, Hodges PW, Refshauge KM, Moseley GL, Herbert RD, Costa LOP, McAuley J (2005) The effect of motor control exercises versus placebo in patients with chronic low back pain. BMC Musculoskelet Disord 6:1–8. doi: 10.1186/1471-2474-6-54)"*
**Participants**	*"From the main study sample (n = 154), a sub-sample of the last 35 participants was selected"*
**Interventions**	*"The objectives of this study were to estimate the reproducibility of ultrasound measures of automatic activation of the lateral abdominal wall muscles during a leg force task in patients with chronic non-specific low back pain"*
**Outcomes**	*"Ultrasound measurements"*
**Notes**	

Risk of bias table

Bias	Authors' judgement	Support for judgement
Random sequence generation (selection bias)	Low risk	*"This study was nested within an existing randomised, blinded, placebo-controlled trial that compared the efficacy of motor control exercise (MCE) versus placebo in patients with chronic non-specific low back pain (Maher et al 2005) The effect of motor control exercises versus placebo in patients with chronic low back pain. BMC Musculoskelet Disord 6:1–8. doi: 10.1186/1471-2474-6-54)"*
Allocation concealment (selection bias)	Low risk	*"The allocation sequence will be generated by author CM. Participants will be scheduled to receive their first treatment within one week of randomisation. "FROM: Maher et al 2005"*
Blinding of participants and personnel (performance bias)	Low risk	*"Participants will be allocated to treatment group using sealed opaque envelopes. "FROM: Maher et al 2005"*
Blinding of outcome assessment (detection bias)	High risk	*Not detailed*
Incomplete outcome data (attrition bias)	High risk	*Not detailed*
Selective reporting (reporting bias)	High risk	*Not detailed*
Other bias	Low risk	*Nothing*

Ferreira 2014

**Methods**	*"A sample of non-specific chronic LBP patients was taken from a randomised controlled trial that compared the efficacy of motor control exercise, general exercise and spinal manipulative therapy. The final 45 subjects to be enrolled in the randomised controlled trial were invited to participate in this study, of whom 34 were*

	eligible to participate"
**Participants**	*"Patients aged between 18 and 80 years with chronic LBP (symptoms for at least 3 months) with or without pain referral to the leg, but without neurological deficit were recruited for the study"*
**Interventions**	*"Based on the randomisation procedure, participants received motor control exercise, general exercise, or spinal manipulative therapy"*
**Outcomes**	*"Clinical outcomes were measured at baseline and after 8 weeks of treatment.*<br><br>*-Global impression of recovery was measured on an 11-point scale.*<br><br>*-Disability was measured using the 24-item version of the Roland Morris disability questionnaire.*<br><br>*-Average pain intensity over the past week was measured on a numerical rating scale.*<br><br>*-Function was measured with a modified patientspecific functional scale"*
**Notes**	

Risk of bias table

Bias	Authors' judgement	Support for judgement
Random sequence generation (selection bias)	Low risk	*"Baseline measures were taken of the two primary outcomes and two secondary outcomes prior to randomization. Subsequently each participant was randomized to a general exercise group, a spinal manipulative therapy group or a motor control exercise group. Randomization was by a random sequence of randomly permuted blocks of sizes 6, 9 and 15"*
Allocation concealment (selection bias)	Low risk	*"....The randomization schedule was known only to one investigator who was not involved in recruiting participants....."*
Blinding of participants and personnel (performance bias)	Low risk	*"......and it was concealed from patients and the other investigators using consecutively numbered, sealed, opaque envelopes"*
Blinding of outcome assessment (detection bias)	Low risk	*"...The randomization schedule was known only to one investigator who was not involved in recruiting participants....."*
Incomplete outcome data (attrition bias)	Low risk	*Outcomes proposed has results*
Selective reporting (reporting bias)	Low risk	*Ok*
Other bias	Low risk	*Nothing*

Gisela Rochade 2015

**Methods**	*"Os procedimentos executados foram aprovados pelo Comitê de Ética em Pesquisa (CAAE: 18352013.3.0000.5208) da Instituição. Todos os voluntários assinaram o Termo de Consentimento Livre e Esclarecido (TCLE), de acordo com a Resolução 466/12. Trata-se de um estudo de intervenção, randomizado e realizado com mulheres jovens, eutróficas, sedentárias e saudáveis"*
**Participants**	*"A amostra foi composta por mulheres entre 18 e 25 anos, foram excluídas àquelas com o IMC fora dos limites de normalidade (maior que 24,9kg/m² e menor que 19,5kg/m²)17, praticantes de atividades físicas nos últimos três meses antes das coletas e portadores de distúrbios neurológicos, articulares ou musculoesqueléticos*

	que pudessem dificultar a execução dos exercícios, ou déficit cognitivo grave, que inviabilizasse o entendimento dos procedimentos da pesquisa"
**Interventions**	*"As voluntárias foram divididas aleatoriamente em dois grupos: no grupo experimental que realizou o método Pilates (grupo GP) e no grupo controle, que foi submetido a uma técnica tradicional de fortalecimento do abdome e de alongamentos estáticos (grupo GC)"*
**Outcomes**	*"A mensuração da espessura dos músculos abdominais foi feita através da distância em milímetros das fáscias superficial e profunda dos músculos transverso abdominal, oblíquo interno, oblíquo externo e reto do abdome, do lado esquerdo da voluntária, repetindo-se três vezes para ser feita a média e sempre ao final da expiração para ser controlada a influência da respiração. A avaliação ultrassonográfica foi realizada através do aparelho HD7, da marca Phillips, com transdutores convexos (C5-2), por um avaliador devidamente treinado. A avaliação da amplitude de movimento ativa da flexão, extensão, rotação e inclinação lateral da coluna torácica e lombar foi realizada através do aparelho flexímetro da marca Sanny"*
**Notes**	

Risk of bias table

Bias	Authors' judgement	Support for judgement
Random sequence generation (selection bias)	Low risk	*"As voluntárias foram divididas aleatoriamente em dois grupos"*
Allocation concealment (selection bias)	High risk	*Not detailed*
Blinding of participants and personnel (performance bias)	High risk	*Can´t be blinded*
Blinding of outcome assessment (detection bias)	High risk	*Not detailed*
Incomplete outcome data (attrition bias)	Low risk	*Detailed in Figure 1 and Tables 1 to 4*
Selective reporting (reporting bias)	Low risk	*Detailed in Figure 1 and Tables 1 to 4*
Other bias	Low risk	*Nothing*

Gong 2016

**Methods**	*"This study was conducted with 30 chronic low back pain patients in their 20–40 s. The selection criterion was mild chronic low back pain patients who could perform activities of daily living and running in place exercise, and those who had any structural abnormality in their spine before participating in the experiment..."*
**Participants**	*"...with 30 chronic low back pain patients in their 20–40 s.*<br><br>*"Training group (n = 15) 27.35 ± 6.16 164.47 ± 8.32 57.70 ± 8.06 M = 2, F = 13"*<br>*"Control group (n = 15) 27.88 ± 6.99 165.00 ± 8.22 59.05 ± 9.96 M = 2, F = 13"*
**Interventions**	*"To maintain the subjects' postures using running in place, a 30 cm width and 30 cm height space was marked to restrict the exercise area and the subjects were instructed to perform the exercise in the center of the area. The subjects straightened their backs and looked straight ahead. They drew their jaws in to a neutral cervical spine position and contracted their transversus abdominis and internal obliques through abdominal drawing-in to maintain a neutral position of the lumbar and the pelvis regions..."*
**Outcomes**	*"Outcome measures: Ultrasonography"*
**Notes**	

Risk of bias table

Bias	Authors' judgement	Support for judgement
Random sequence generation (selection bias)	Low risk	*"The subjects were randomly assigned by computer to a training group that would participate in running in place and a control group that would not participate in running in place"*
Allocation concealment (selection bias)	High risk	*Can´t be blinded.*
Blinding of participants and personnel (performance bias)	High risk	*Not detailed.*
Blinding of outcome assessment (detection bias)	High risk	*Not detailed.*
Incomplete outcome data (attrition bias)	Low risk	*All variables has data.*
Selective reporting (reporting bias)	Low risk	*Ok.*
Other bias	Low risk	*Nothing*

Guthrie 2012

**Methods**	*"To investigate the ability of 2 types of bridging-exercise progressions to facilitate lateral abdominal muscles during an abdominal drawing-in maneuver (ADIM) in individuals with LBP. Design: Randomized control trial. Setting: University research laboratory"*
**Participants**	*"Fifty-one adults (18 men, 33 women) with a current episode of LBP participated in this study. Participants were recruited from the university community and from an athletic therapy clinic. Inclusion criteria were based on physical examination and history findings consistent with the stabilization classification, which is a component of a treatment-based classification system for individuals with LBP"*
**Interventions**	*"Participants were verbally instructed to gently pull their navel to the spine at the end of a normal exhalation and to hold this contraction for 10 seconds while continuing normal respiration. During the training session, the researcher monitored participant progress using ultrasound imaging but did not allow the participant to visualize the ultrasound screen......the traditional-bridging group or the suspension-exercise bridging group"*
**Outcomes**	*"Thicknesses of the EO, IO, and TrA were measured using Image J software"*
**Notes**	

Risk of bias table

Bias	Authors' judgement	Support for judgement
Random sequence generation (selection bias)	Low risk	*2The subjects were randomly assigned by computer to a training group that would participate in running in place and a control group that would not participate in running in place"*
Allocation concealment (selection bias)	Low risk	*Can´t be blinded.*
Blinding of participants and personnel (performance bias)	Low risk	*Not detailed.*
Blinding of outcome assessment (detection bias)	Low risk	*Not detailed.*
Incomplete outcome data (attrition bias)	Low risk	*All variables has data.*
Selective reporting (reporting bias)	Low risk	*Ok.*

Other bias	Low risk	*Nothing*

Halliday 2016

**Methods**	*"This study was a randomized, assessor-blinded, clinical trial with an eight week follow-up. Ethics approval was granted by the Sydney Local Health District Human Ethics Committee. The study was registered retrospectively with the Australian New Zealand Clinical Trials Registry, trial number CTRN12611000971932"*
**Participants**	*"Diagnosis: People with chronic low back pain (LBP) classified with a directional preference N= 70 Age: Average age 48.8 Sex: Males and Females. Inclusion: a greater than 3-month history 125 of LBP and a directional preference observed with a mechanical assessment based on the McKenzie method. The area of pain could be localized between the twelfth rib and the buttock crease. Patients reporting referred pain into one or both legs with or without sensory and or motor changes were also included"*
**Interventions**	*"Mckenzie Method vs Motor control Exercise"*
**Outcomes**	*"All outcomes were collected at baseline and at 8-week follow-up by blinded assessors. The primary outcome measurement was recruitment of the trunk muscles TrA, obliquus externus (OE) and obliquus internus (OI) expressed as percentage changes in muscle thickness increases from base line to eight week follow-up. Measurements of trunk muscle thickness were obtained from real time ultrasound images"*
**Notes**	

Risk of bias table

Bias	Authors' judgement	Support for judgement
Random sequence generation (selection bias)	Low risk	*"The randomization sequence was created using computer generated numbers by a researcher not involved with data collection"*
Allocation concealment (selection bias)	Low risk	*"Following baseline data collection patients were randomized to treatment allocation by a research assistant who was unaware of the randomization sequence. This process was conducted.by opening sequentially numbered, opaque sealed envelopes"*
Blinding of participants and personnel (performance bias)	Unclear risk	*Not clear.*
Blinding of outcome assessment (detection bias)	Low risk	*"The research assistant responsible for the collection of ultrasound images was blinded to group Allocation"*
Incomplete outcome data (attrition bias)	Low risk	*"Reasons for being unavailable for data collection included: time constraints [n=5], dissatisfaction with treatment [n=2], and inability to attend treatment sessions [n=1]"*
Selective reporting (reporting bias)	Low risk	*"There was no statistically significant 323 difference between treatment groups on the recruitment of the three abdominal muscles"*
Other bias	Low risk	*Nothing.*

Hoppes 2016

**Methods**	*"Randomized controlled trial. The study protocol was approved by the Institutional*

	Review Board of Brooke Army Medical Center. All participants provided informed consent prior to study enrollment"
**Participants**	*"Participants were all active duty U.S. service members who responded to recruiting advertisements in the Army Medical Department Center and School at Joint Base San Antonio, Texas. Study inclusion criteria consisted of greater than 18 years of age, able to perform standard physical training, and no conditions that may have affected standing balance. Exclusion criteria included presence of low back pain and inability to perform the prescribed core stability regimen. N=33"*
**Interventions**	*"An eight-week core stability exercise program would result in a larger improvement in physical endurance and abdominal muscle thickness than a control intervention"*
**Outcomes**	*"Ultrasound imaging was used to measure the muscle thickness of the transversus abdominis (TrA) and internal oblique (IO) at rest and with the TrA preferentially contracted"*
**Notes**	

Risk of bias table

Bias	Authors' judgement	Support for judgement
Random sequence generation (selection bias)	Low risk	*"Participants were randomized into either the core strengthening exercise group or the control group"*
Allocation concealment (selection bias)	Low risk	*"...and allocation concealment was preserved until the moment of group assignment"*
Blinding of participants and personnel (performance bias)	High risk	*Can´t be blinded*
Blinding of outcome assessment (detection bias)	High risk	*Not detailed*
Incomplete outcome data (attrition bias)	Low risk	*Detailed in tables 1 and 2 and figures 2 to 4.*
Selective reporting (reporting bias)	Low risk	*Detailed in tables 1 and 2 and figures 2 to 4.*
Other bias	Low risk	*Nothing*

Nabavi 2017

**Methods**	*"A randomized controlled trial was designed to compare the effect of stabilization versus routine exercises on pain intensity and muscle dimensions in patients with nonspecific chronic LPB"*
**Participants**	*"Forty-one patients with nonspecific chronic LBP, who were referred by an orthopedic surgeon, were recruited in an outpatient orthopedic clinic. The participants were included if they were between 18 and 55 years old, had good general health (using the Farsi version of the 12-itemGeneral Health Questionnaire). (n = 20) receiving electrotherapy and stabilization exercises or a control group (n = 21) receiving electrotherapy and routine exercises"*
**Interventions**	*"Both groups received routine physiotherapy including electrotherapy and warmup exercises. Electrotherapy is routinely prescribed by different consultants for the physiotherapy treatment of patients with LBP"*
**Outcomes**	*"The patients then completed a questionnaire including questions on demographic*

	data such as gender, weight, height, body mass index, history of back pain, and general health condition and pain intensity was assessed on a visual analog scale. This was followed by measuring muscle dimensions (right and left TrA and MF muscles) by a radiologist using US"
**Notes**	

Risk of bias table

Bias	Authors' judgement	Support for judgement
Random sequence generation (selection bias)	Low risk	*"Patients were randomly assigned (using a block-style randomization scheme)"*
Allocation concealment (selection bias)	High risk	*Not detailed*
Blinding of participants and personnel (performance bias)	High risk	*Not detailed*
Blinding of outcome assessment (detection bias)	Low risk	*Tables 1 and 2*
Incomplete outcome data (attrition bias)	Low risk	*Table 1 and 2*
Selective reporting (reporting bias)	High risk	*Not detailed*
Other bias	Low risk	*ok*

Shamsi 2016

**Methods**	*"A quasi-randomized controlled trial was conducted (Trial Registration number: IRCT201111098035N1). Approval for the research was received from the ethics committee of Iran University of Medical Sciences (IUMS)"*
**Participants**	*"Forty-eight non-specific CLBP patients enrolled in the present study. Inclusion criteria were: (1) having LBP for more than 3 months; (2) pain intensity from 3 to 6 on the visual analogue scale (VAS scale); and (3) age of 18 to 60 years"*
**Interventions**	*"Both programs had a common component of warm-up (eight stretching exercises and stationary bicycling for 5 minutes). Based on previous recommendations two programs with eight stages were performed (Koumantakis, Watson, and Oldham, 2005). The difficulty of exercises was increased progressively in each stage. An explanation on how to perform the exercises was provided to the participants in the first session"*
**Outcomes**	*"Outcome measures: Ultrasound measurement of the abdominal muscle thickness, Disability and pain"*
**Notes**	

Risk of bias table

Bias	Authors' judgement	Support for judgement
Random sequence generation (selection bias)	Unclear risk	*"At the time of admission, participants were assigned a number in the order that they entered the study. Those with odd numbers were selected to the core stability exercise (CSE) group and those with even numbers to the general exercise (GE) Group"*
Allocation concealment (selection bias)	High risk	*Not detailed.*
Blinding of participants and personnel (performance bias)	High risk	*Not detailed.*

Blinding of outcome assessment (detection bias)	High risk	*Not detailed.*
Incomplete outcome data (attrition bias)	Low risk	*Table 2 and 3*
Selective reporting (reporting bias)	Low risk	*Table 2 and 3*
Other bias	Low risk	*ok*

Tajiri 2014

**Methods**	*"The purpose of this study was to devise a new urinary incontinence exercise using co-contraction of both the transverse abdominal muscle (TA) and pelvic floor muscle (PFM) and examine the intervention effect in middle-aged women with stress urinary incontinence (SUI)"*
**Participants**	*"The subjects were fifteen women who had experienced one or more SUI events in the past 1 month. The subjects were divided into two groups randomly: the TA and PFM co-contraction exercise group (n=9) and the control group (n=6)"*
**Interventions**	*"Subjects in the exercise group were provided with an 8-week TA and PFM co-contraction training program. The exercises prescribed were 40 repetitions (2 sets of 20 repetitions) of a 3-second co-contraction of both the TA and PFM. The women were told to perform 1 session of exercise 3 times per week. The women in the control group were asked not to exercise at home during the study but were offered the possibility of receiving a treatment at trial completion"*
**Outcomes**	*"All subjects completed a questionnaire about SUI. We evaluated the thickness of the TA using ultrasound. The thickness of the TA was measured in all subjects under four conditions at random in the supine position. 1) The first condition was the resting state. 2) The second condition was maximal contraction of the TA. For this, the subjects were instructed to draw in the lower abdominal wall toward the spine, an action that specifically activates the TA. The subjects were asked to breathe in a relaxed manner. No movement of the lumbar spine was allowed. 3) The third condition was maximal contraction of the PFM. For this the subjects were instructed to contract the muscles around the vagina "like a drawstring" and to lift them internally. No posterior tilt of the pelvis was allowed. There was no instruction to either use or not use the abdominal muscles. 4) The fourth condition was maximal co-contraction of both the TA and PFM. Subjects were instructed to draw in the lower abdominal wall toward the spine, an action that specifically activates the TA. When the TA sustained isometric contraction, the subjects were instructed to contract the muscles around the vagina "like a drawstring" to lift them internally and to keep this position for 3 seconds"*
**Notes**	

Risk of bias table

Bias	Authors' judgement	Support for judgement
Random sequence generation (selection bias)	Low risk	*"The subjects were divided into two groups randomly"*
Allocation concealment (selection bias)	High risk	*Not detailed.*
Blinding of participants and personnel (performance bias)	High risk	*Not detailed.*
Blinding of outcome assessment (detection bias)	High risk	*Not detailed.*
Incomplete outcome data (attrition bias)	Low risk	*Tables 1 y 2*
Selective reporting (reporting bias)	Low risk	*Tables 1 y 2*
Other bias	Low risk	*nothing*

Teyhen 2005

**Methods**	*"Randomized controlled trial among patients with low back pain (LBP). (1) Determine the reliability of real-time ultrasound imaging for assessing activation of the lateral abdominal muscles; (2) characterize the extent to which the abdominal drawing-in maneuver (ADIM) results in preferential activation of the transverse abdominis (TrA); and (3) determine if ultrasound biofeedback improves short-term performance of the ADIM in patients with LBP"*
**Participants**	*"A convenience sample of 30 subjects (12 women) was recruited by physical therapists from 2military medical centers (Brooke Army Medical Center and Wilford Hall Medical Center) in San Antonio, TX. The study was approved by the joint Brooke Army Medical Center and Wilford Hall Medical Center Institutional Review Board. All subjects provided consent to their participation and the rights of the subjects were protected"*
**Interventions**	*"...lumbar stabilization training were randomized to receive either traditional training (n = 15) or traditional training with biofeedback (n = 15)"*
**Outcomes**	*"Ultrasound Measurements Ultrasound measurements were obtained with the subject in the supine hooklying position and the examiners on the left side of the subject"*
**Notes**	

Risk of bias table

Bias	Authors' judgement	Support for judgement
Random sequence generation (selection bias)	Low risk	*"Randomized controlled trial among patients with low back pain"*
Allocation concealment (selection bias)	Low risk	*"After the training in quadruped, patients were then randomly assigned to receive further instruction using traditional training or traditional training with biofeedback in the ADIM. The randomization scheme was performed prior to the initiation of the study, using sealed, sequentially numbered envelopes that corresponded to the patient's study identification number"*
Blinding of participants and personnel (performance bias)	Low risk	*"To minimize bias, a team of 2 examiners performed the ultrasound measurements. One examiner positioned the transducer and optimized the quality of the image, but was blinded to the actual measurement values. A second examiner blinded to group assignment recorded the results of all measurements"*
Blinding of outcome assessment (detection bias)	Low risk	*"Although all repeated measures were recorded by the same examiner, the potential for recall bias was controlled by blinding the examiner to the results of each measurement"*
Incomplete outcome data (attrition bias)	Low risk	*Tables 1, 2, and 3*
Selective reporting (reporting bias)	Low risk	*Tables 4 and 5, Figure 3.*
Other bias	Low risk	*Nothing.*

Vasseljen 2010

**Methods**	*"Subjects with LBP were recruited from local medical practitioners and through advertisement. The study was approved by the Regional Ethics Committee and is a sub-study of a registered clinical trial (clinicaltrials.gov: NCT00201513). Participants*

	gave signed consent after receiving verbal and written information about the study"
**Participants**	*"Patients (n = 109) were randomized to specific ultrasound guided, sling or general exercises. Men and women at age 18-60 years with non-specific chronic LBP (>12 weeks) and pain at presentation between 2 and 8 on an 11-point Numeric Rating Scale (NRS 0e10) were included"*
**Interventions**	*"...8 weeks of exercise in chronic low back pain patients"*
**Outcomes**	*"Ultrasound recordings during ADIM test and pain"*
**Notes**	

Risk of bias table

Bias	Authors' judgement	Support for judgement
Random sequence generation (selection bias)	Low risk	*"Subjects in this RCT study were randomly assigned to either low load ultrasound guided ADIM exercises, high load sling exercises or general exercises"*
Allocation concealment (selection bias)	Low risk	*2Block randomization with a random sequence of permuted blocks of variable sizes from 3 to 9 was used and administered by an independent project secretary......Ultrasound measurements of thickness and slide were performed by a person blind to group allocation"*
Blinding of participants and personnel (performance bias)	Low risk	*"Ultrasound measurements of thickness and slide were performed by a person blind to group allocation"*
Blinding of outcome assessment (detection bias)	High risk	*Not detailed.*
Incomplete outcome data (attrition bias)	Low risk	*Table 1 to 4 and Figure 4-5*
Selective reporting (reporting bias)	Low risk	*Table 1 to 4 and Figure 4-5*
Other bias	Low risk	*Nothing*

Worth 2007

**Methods**	*"We aimed to recruit a cross sectional sample of 20 subjects with LBP from a local physiotherapy clinic. Volunteers were excluded if they had: 1) spinal surgery; 2) spinal deformities; 3) known neuromuscular or joint disease; 4) a history of cancer, or, 5) if they were pregnant. Any volunteers who had any prior experience with the AHE were also excluded from the study. Each volunteer gave his/her informed consent by signing a lay summary and consent form approved by the University of Vermont's Institutional Review Boar"*
**Participants**	*"Nineteen patients with low back pain were randomly divided into two feedback groups"*
**Interventions**	*"Group 1 received typical clinical instructicn whilst attempting the abdominal hcllcwing exercise, whereas Group 2 additionally received visual feedback frcm the ultrascund image"*
**Outcomes**	*"1) an observable thickening and lateral movement of the TA muscle and thickening ofthe IO muscle, which was verified by imaging the anterolateral abdominal wall with RTUS; 2) no contraction of the EO muscle, which was verified by an absence of muscle thickening on the RTUS image and by*

	psdpation of this muscle by the physiotherapist; 3) minimal to no movement ofthe pelvis in the posterior direction, which was verified by visual inspection and palpation of the pelvis; 4) no increased weight bearing through the subjects' heels, which was verified by visual inspection; and (5) no deep inspiration followed and clinician determined by visual inspection and palpation of the anterior thorax, McGill Pain Questionnaire, Oswestry Disability Scale, and Numeric Pain Index"
**Notes**	

Risk of bias table

Bias	Authors' judgement	Support for judgement
Random sequence generation (selection bias)	Low risk	*"......were randomly divided into two feedback groups"*
Allocation concealment (selection bias)	High risk	*Not detailed.*
Blinding of participants and personnel (performance bias)	High risk	*Not detailed.*
Blinding of outcome assessment (detection bias)	High risk	*Not detailed.*
Incomplete outcome data (attrition bias)	Low risk	*"Tables 1,2,3 and 4 & Figures 1 and 2"*
Selective reporting (reporting bias)	Low risk	*"Tables 1,2,3 and 4 & Figures 1 and 2"*
Other bias	Low risk	*Nothing*

PELVIC FLOOR

Bernardes 2012

**Methods**	*"Single-blind randomized controlled trial. All participants received a three-month intervention according to their group allocation after the first evaluation, and received a second evaluation after the intervention period"*
**Participants**	*"58 women with pelvic organ prolapse who were patients at the Urogynecology and Vaginal Surgery outpatient clinic of Universidade Federal de São Paulo were evaluated by a gynecologist during a routine consultation and were asked to participate"*
**Interventions**	*"Two treatment groups, with 21 patients each, consisting of PFM training (Group I [GI]), hypopressive exercises plus voluntary pelvic floor muscle contraction (Group II [GII])"*
**Outcomes**	*"We then evaluated the CSA of the levator ani muscle using two-dimensional transperineal ultrasonography"*
**Notes**	

Risk of bias table

Bias	Authors' judgement	Support for judgement
Random sequence generation (selection bias)	Low risk	The group allocations were undertaken using computer- generated random numbers to stratify the randomization
Allocation concealment (selection bias)	Low risk	The main investigation was blind to the group allocation.
Blinding of participants and personnel (performance bias)	Unclear risk	Not detailed

Blinding of outcome assessment (detection bias)	Unclear risk	Not clear were was the blinding.
Incomplete outcome data (attrition bias)	Low risk	All data proposed available.
Selective reporting (reporting bias)	Low risk	Same before
Other bias	Low risk	Nothing

Johannesen 2016

**Methods**	*"A two-armed randomised controlled trial was conducted at Østfold Hospital Trust and St Olavs Hospital in Norway during 2010–2014"*
**Participants**	*"The study population consisted of parous Norwegian-speaking women over the age of 18 who reported AI on inclusion (fecal incontinence monthly or more often or St. Mark's score ≥3 points).10 The study included women with and without obstetrical anal sphincter injury (OASI) and primary sphincter repair. Exclusion criteria were inadequate Norwegian language skills, neurological conditions such as multiple sclerosis and polio, women already receiving PFME treatment before inclusion due to severe postpartum AI or pelvic floor dysfunction, PFM pain/dysfunction, secondary sphincter repair, and being unable to attend treatment and follow up at the nearest available anorectal out-patient clinic or community pelvic floor physiotherapist. N=109"*
**Interventions**	*"The intervention group (PFME group) received 6 months of individual physiotherapy-guided PFME and the control group received written information on PFME only"*
**Outcomes**	*"The primary outcome measure was change in AI from baseline to post-intervention, as measured on the St. Mark's score.....At Østfold Hospital Trust, two-dimensional (2D) and threedimensional EAUS was performed in a side-lying position using a B-K Medical EAUS machine type 2050 with a 360° probe (B-K Medical Aps, Denmark). At St Olavs Hospital, 2D imaging was performed in the supine position using a Hitachi Hi-Vision EAUS machine (Hitachi EUB-6500, Providian Medical Equipment, USA)"*
**Notes**	*"Nothing"*

Risk of bias table

Bias	Authors' judgement	Support for judgement
Random sequence generation (selection bias)	Low risk	The randomisation was performed using an internetbased computerised procedure at the Unit for Applied Clinical Research at the Norwegian University of Science and Technology, Norway
Allocation concealment (selection bias)	High risk	Not detailed
Blinding of participants and personnel (performance bias)	High risk	Assessors and participants were not blinded due to the nature of the study and financial restraints.
Blinding of outcome assessment (detection bias)	High risk	Not detailed
Incomplete outcome data (attrition bias)	Low risk	Detailed in Figure 1 and Tables 1 to 3
Selective reporting (reporting bias)	Low risk	Detailed in Figure 1 and Tables 1 to 3
Other bias	Low risk	Nothing

McLean 2013

**Methods**	*"The purpose of this study was to investigate the impact of a physiotherapist-supervised 12-week PFM training program for women with SUI on resting bladder neck position, bladder neck mobility during coughing and Valsalva tasks, and on urethral morphology. This study was approved by the Queen's University and Affiliated Hospitals Health Sciences Research Ethics Board, and all women provided written informed consent prior to participating"*
**Participants**	*"Forty women with SUI were randomly assigned to one of two groups"*
**Interventions**	*"The treatment group received 12 weekly physiotherapy sessions during which they learned how to properly contract their pelvic floor muscles (PFMs) and a home exercise program was prescribed, reviewed, and progressed; the control group received no treatment"*
**Outcomes**	*"Before and after the 12-week study period, ultrasound imaging was used to evaluate bladder neck position and mobility during coughing and Valsalva maneuver in supine and in standing, as well as urethral morphology. Secondary outcome measures included a 3-day bladder diary, 30-min pad test, the Incontinence Impact Questionnaire (IIQ-7) and the Urogenital Distress Inventory (UDI-6)"*
**Notes**	

Risk of bias table

Bias	Authors' judgement	Support for judgement
Random sequence generation (selection bias)	Low risk	20 of these were randomly allocated to the intervention cohort and 20 were allocated to the control cohort using a custom automated computer algorithm.
Allocation concealment (selection bias)	High risk	Not blinded
Blinding of participants and personnel (performance bias)	High risk	Not blinded.
Blinding of outcome assessment (detection bias)	High risk	Not blinded.
Incomplete outcome data (attrition bias)	Low risk	All data outcomes data available.
Selective reporting (reporting bias)	Low risk	All data available.
Other bias	Low risk	Nothing

Stuge 2006

**Methods**	*"The aim was to examine whether subjects with and without persisting Pelvic Girdle Pain (PGP) and disability, independent of the preceding treatment, differed with respect to the ability to voluntarily contract the deep abdominal muscles (TrA and IO) and to the strength of the PFM"*
**Participants**	*"Women with PGP who had participated in a randomized controlled trial (n= 81) with a 2-year follow-up study, evaluating the effect of two different physical therapy interventions to treat postpartum PGP"*
**Interventions**	*"Contractions of the deep abdominals, Pelvic floor muscle (PFM) contraction"*
**Outcomes**	*"The women completed a short questionnaire addressing weight, height, pain location (VAS scale), functional status (Disability was measured by Roland-Morris Disability Questionnaire)(Pelvic floor muscle contraction: Vaginal observation and palpation) (Measurement of pelvic floor muscle strength: A vaginal balloon catheter (balloon size 6.71.7 cm) connected to a pressure*

	transducer), symptoms of urinary incontinence and other pelvic floor complaints, physical activity level and age of youngest child"
**Notes**	

Risk of bias table

Bias	Authors' judgement	Support for judgement
Random sequence generation (selection bias)	High risk	The subjects were also categorized as ASLR positive or ASLR negative.
Allocation concealment (selection bias)	High risk	Not detailed.
Blinding of participants and personnel (performance bias)	Low risk	The two researchers (SM, HHD) performing the assessments were blinded to the patients' symptoms, history of treatment and the results of the other assessor's assessments.
Blinding of outcome assessment (detection bias)	Low risk	Same before.
Incomplete outcome data (attrition bias)	Low risk	All data
Selective reporting (reporting bias)	Low risk	All data
Other bias	Low risk	Nothing

Tajiri 2014

**Methods**	*"The purpose of this study was to devise a new urinary incontinence exercise using cocontraction of both the transverse abdominal muscle (TA) and pelvic floor muscle (PFM) and examine the intervention effect in middle-aged women with stress urinary incontinence (SUI)"*
**Participants**	*"The subjects were fifteen women who had experienced one or more SUI events in the past 1 month. The subjects were divided into two groups randomly: the TA and PFM co-contraction exercise group (n=9) and the control group (n=6)"*
**Interventions**	*"Subjects in the exercise group were provided with an 8-week TA and PFM co-contraction training program. The exercises prescribed were 40 repetitions (2 sets of 20 repetitions) of a 3-second co-contraction of both the TA and PFM. The women were told to perform 1 session of exercise 3 times per week. The women in the control group were asked not to exercise at home during the study but were offered the possibility of receiving a treatment at trial completion"*
**Outcomes**	*"All subjects completed a questionnaire about SUI. We evaluated the thickness of the TA using ultrasound. The thickness of the TA was measured in all subjects under four conditions at random in the supine position. 1) The first condition was the resting state. 2) The second condition was maximal contraction of the TA. For this, the subjects were instructed to draw in the lower abdominal wall toward the spine, an action that specifically activates the TA. The subjects were asked to breathe in a relaxed manner. No movement of the lumbar spine was allowed. 3) The third condition was maximal contraction of the PFM. For this the subjects were instructed to contract the muscles around the vagina "like a drawstring" and to lift them internally. No posterior tilt of the pelvis was allowed. There was no instruction to either use or not use the abdominal muscles. 4) The fourth condition was maximal co-contraction of both the TA and PFM. Subjects were instructed to draw in the lower abdominal wall toward the spine, an action that specifically activates the TA. When the TA sustained isometric contraction, the subjects were instructed to contract the muscles around the vagina "like a drawstring" to lift them internally and to keep this position for 3 seconds"*
**Notes**	

Risk of bias table

Bias	Authors' judgement	Support for judgement
Random sequence generation (selection bias)	Low risk	The subjects were divided into two groups randomly:
Allocation concealment (selection bias)	High risk	Not detailed.
Blinding of participants and personnel (performance bias)	High risk	Not detailed.
Blinding of outcome assessment (detection bias)	High risk	Not detailed.
Incomplete outcome data (attrition bias)	Low risk	Table 2
Selective reporting (reporting bias)	Low risk	Table 2
Other bias	Low risk	Nothing.

Higgins JPT, Green S (editors). *Cochrane Handbook for Systematic Reviews of Interventions* Version 5.1.0 [updated March 2011]. The Cochrane Collaboration, 2011. Available from www.handbook.cochrane.org.

Supplementary File S4: Complete annotated Forest plot-graphs and tables.

Fernández-Carnero S, Martin-Saborido C, Achalandabaso Ochoa-Ruiz de Mendoza A, Ferragut-Garcias A, Cuenca-Zaldívar JN, Leal-Quiñones A, Calvo-Lobo C, Gallego-Izquierdo T

The role of Rehabilitative Ultrasound Imaging Technique in lumbopelvic region as a diagnosis and treatment tool in Physiotherapy. Systematic Review, meta-analisis and meta-regression.

Complete annotated forest plots-graphs

LUMBAR

Graph S7 Multifidus muscles thickness forest plot

Study or Subgroup	Favours [Control Exercis] Mean	SD	Total	Control Mean	SD	Total	Weight	Mean Difference IV, Fixed, 95% CI
Akbari 2008	−1.06	0.69	25	−0.43	0.45	24	3.2%	−0.63 [−0.95, −0.31]
Berglund 2017	0.02	0.11	33	−0.08	0.13	32	96.8%	0.10 [0.04, 0.16]
**Total (95% CI)**			**58**			**56**	**100.0%**	**0.08 [0.02, 0.13]**

Heterogeneity: $Chi^2 = 18.78, df = 1 (P < 0.0001); I^2 = 95\%$
Test for overall effect: Z = 2.62 (P = 0.009)

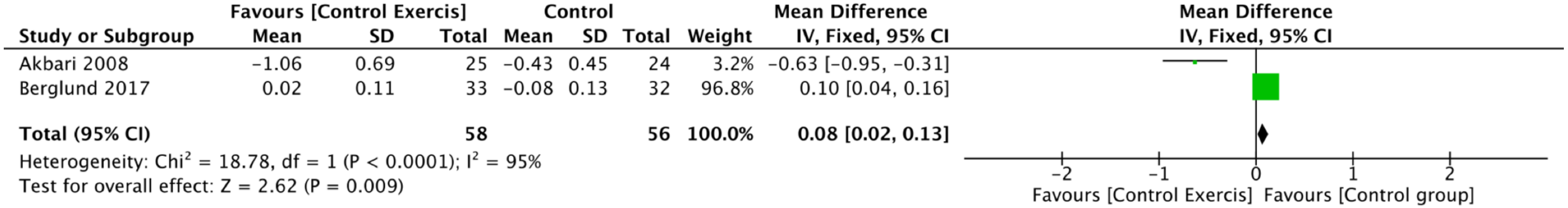

Graph S8 Pain in lumbar regions forest plot

Study or Subgroup	Experimental Mean	SD	Total	Control Mean	SD	Total	Weight	Mean Difference IV, Fixed, 95% CI
Akbari 2008	−4.75	0.315	25	−4	0.4	24	97.7%	−0.75 [−0.95, −0.55]
Berglund 2017	−1.8	2.67	31	−1.9	2.55	29	2.3%	0.10 [−1.22, 1.42]
**Total (95% CI)**			**56**			**53**	**100.0%**	**−0.73 [−0.93, −0.53]**

Heterogeneity: $Chi^2 = 1.55, df = 1 (P = 0.21); I^2 = 36\%$
Test for overall effect: Z = 7.17 (P < 0.00001)

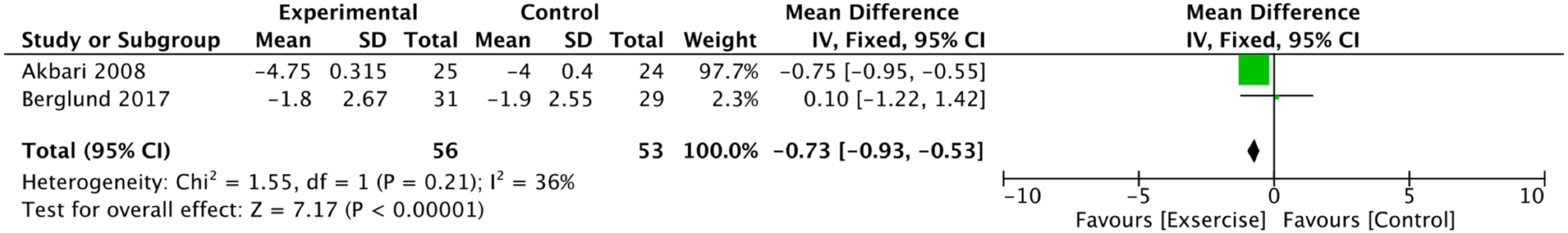

ABDOMINAL

Graph S9 Pain in abdominal region forest plot

	Exercise Group			Control Group				Mean Difference
Study or Subgroup	Mean	SD	Total	Mean	SD	Total	Weight	IV, Fixed, 95% CI
Ferreira 2014	2.36	3.2	11	2.8	2.7	10	0.4%	−0.44 [−2.97, 2.09]
Halliday 2016	2.9	0.53	35	2.1	0.53	34	39.4%	0.80 [0.55, 1.05]
Nabavi 2017	3.3	2.69	21	3.48	2.97	20	0.8%	−0.18 [−1.92, 1.56]
Shamsi 2016	3.6	0.32	22	3.8	0.36	21	59.3%	−0.20 [−0.40, 0.00]
**Total (95% CI)**			**89**			**85**	**100.0%**	**0.19 [0.04, 0.35]**

Heterogeneity: $Chi^2 = 37.31$, df = 3 ($P < 0.00001$); $I^2 = 92\%$
Test for overall effect: Z = 2.42 (P = 0.02)

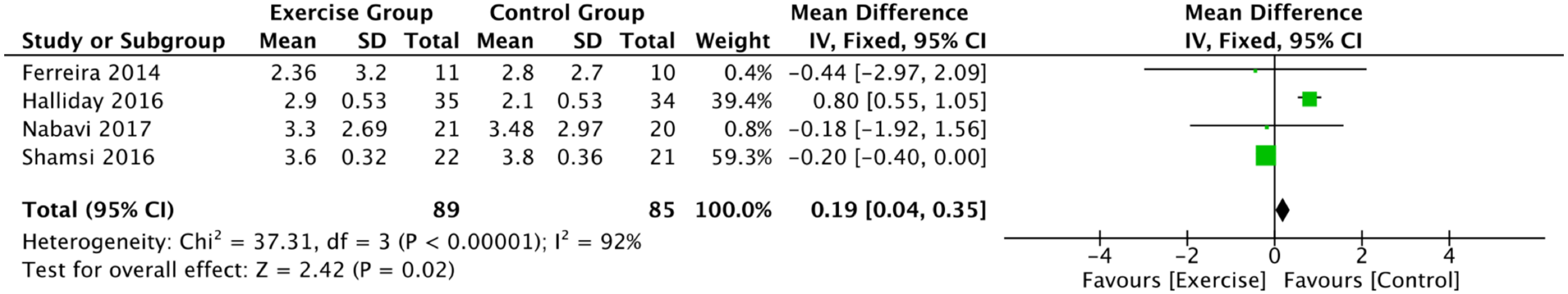

Graph S10 Transversus abdominis muscles thickness forest plot

	Exercise Group			Control Group				Mean Difference
Study or Subgroup	Mean	SD	Total	Mean	SD	Total	Weight	IV, Fixed, 95% CI
**2.1.1 Muscle thicness of TrA in %**								
Ferreira 2014	7.8	12.8	11	8.8	12.1	10	0.0%	−1.00 [−11.65, 9.65]
Halliday 2016	3.06	5.68	35	−2.25	5.02	34	0.3%	5.31 [2.78, 7.84]
**Subtotal (95% CI)**			**46**			**44**	**0.4%**	**4.97 [2.51, 7.43]**
Heterogeneity: $Chi^2 = 1.28$, df = 1 (P = 0.26); $I^2 = 22\%$								
Test for overall effect: Z = 3.96 ($P < 0.0001$)								
**2.1.2 Muscle Thickness as Mean Difference**								
Nabavi 2017	0.48	0.25	20	0.37	0.56	21	31.1%	0.11 [−0.15, 0.37]
Shamsi 2016	0.01	0.36	22	0.19	0.22	21	68.5%	−0.18 [−0.36, −0.00]
**Subtotal (95% CI)**			**42**			**42**	**99.6%**	**−0.09 [−0.24, 0.06]**
Heterogeneity: $Chi^2 = 3.20$, df = 1 (P = 0.07); $I^2 = 69\%$								
Test for overall effect: Z = 1.19 (P = 0.23)								
**Total (95% CI)**			**88**			**86**	**100.0%**	**−0.07 [−0.22, 0.08]**

Heterogeneity: $Chi^2 = 20.70$, df = 3 (P = 0.0001); $I^2 = 86\%$
Test for overall effect: Z = 0.95 (P = 0.34)
Test for subgroup differences: $Chi^2 = 16.23$, df = 1 ($P < 0.0001$), $I^2 = 93.8\%$

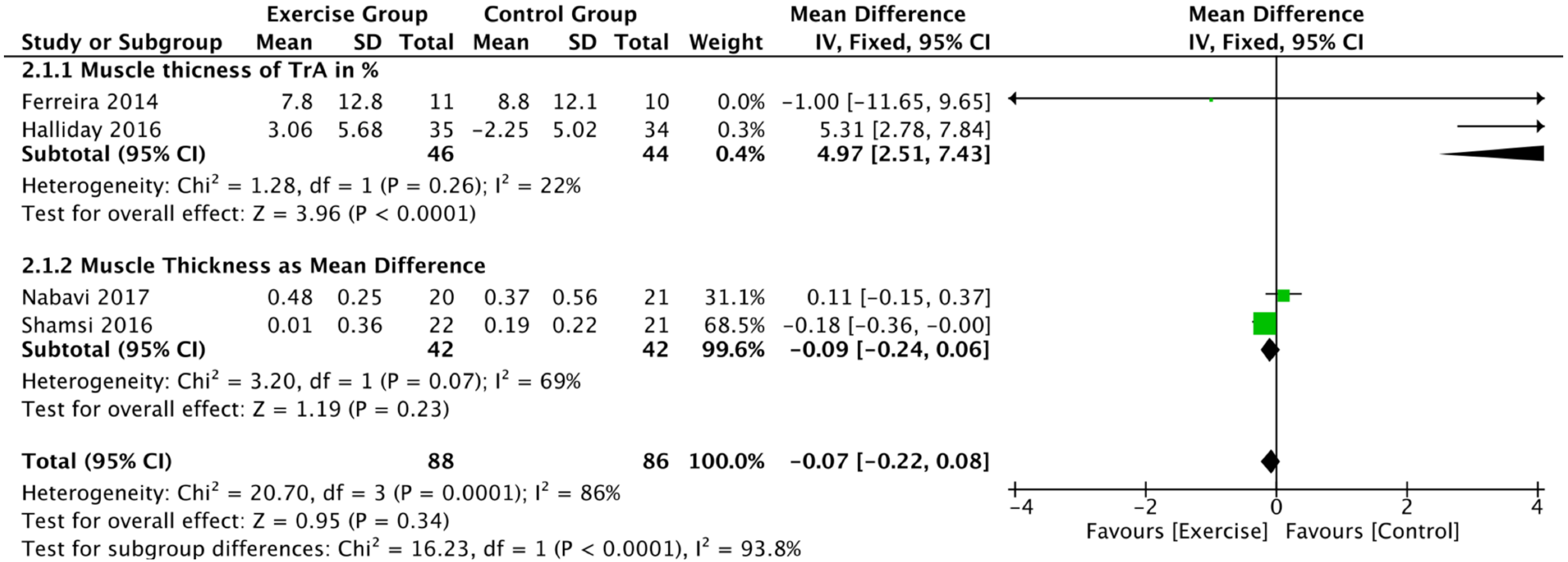

Graph S11 Internal oblique muscles thickness forest plot

Study or Subgroup	Exercise Group Mean	SD	Total	Control Group Mean	SD	Total	Weight	Mean Difference IV, Fixed, 95% CI
Halliday 2016	7.79	12.92	35	7.5	12.92	34	3.4%	0.29 [−5.81, 6.39]
Shamsi 2016	5.85	2	22	5.88	1.8	21	96.6%	−0.03 [−1.17, 1.11]
**Total (95% CI)**			**57**			**55**	**100.0%**	**−0.02 [−1.14, 1.10]**

Heterogeneity: $Chi^2 = 0.01$, df = 1 (P = 0.92); $I^2 = 0\%$

Test for overall effect: Z = 0.03 (P = 0.97)

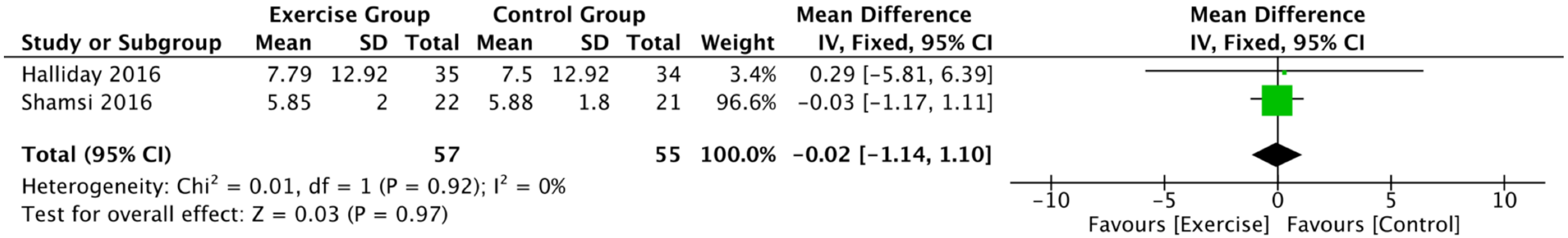

Graph S12 External oblique muscles thickness forest plot

Study or Subgroup	Exercise Group Mean	SD	Total	Control Group Mean	SD	Total	Weight	Mean Difference IV, Fixed, 95% CI
Halliday 2016	5.47	11.41	35	3.21	11.82	34	1.8%	2.26 [−3.22, 7.74]
Shamsi 2016	4.83	1.3	22	4.77	1.2	21	98.2%	0.06 [−0.69, 0.81]
**Total (95% CI)**			**57**			**55**	**100.0%**	**0.10 [−0.64, 0.84]**

Heterogeneity: $Chi^2 = 0.61$, df = 1 (P = 0.44); $I^2 = 0\%$

Test for overall effect: Z = 0.26 (P = 0.79)

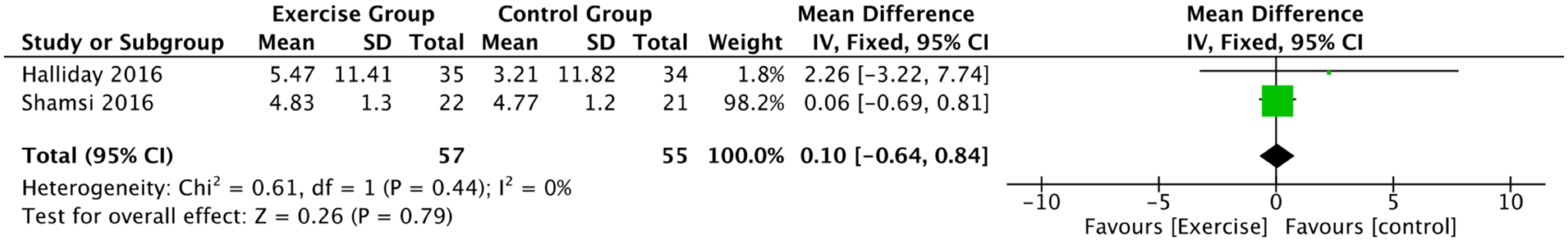

Complete annotated forest plots-tables

Table S3 Summary of lumbar muscle thickness

Outcome or Subgroup	Studies	Participants	Statistical Method	Effect Estimate
1.2 Muscle Thickness	2	114	Mean Difference (IV, Fixed, 95% CI)	0.08 [0.02, 0.13]

Table S4 Summary of lumbar pain

Outcome or Subgroup	Studies	Participants	Statistical Method	Effect Estimate
2.1 PAIN	2	109	Mean Difference (IV, Fixed, 95% CI)	-0.73 [-0.93, -0.53]

Table S5 Summary of abdominal pain.

Outcome or Subgroup	Studies	Participants	Statistical Method	Effect Estimate
3.1 Pain	4	174	Mean Difference (IV, Fixed, 95% CI)	0.19 [0.04, 0.35]

Table S6 Summary of TrA muscle thickness.

Outcome or Subgroup	Studies	Participants	Statistical Method	Effect Estimate
4.1 Muscles thickness of TrA	4	174	Mean Difference (IV, Fixed, 95% CI)	-0.07 [-0.22, 0.08]
4.1.1 Muscle thickness of TrA in %	2	90	Mean Difference (IV, Fixed, 95% CI)	4.97 [2.51, 7.43]
4.1.2 Muscle thickness as Mean Difference	2	84	Mean Difference (IV, Fixed, 95% CI)	-0.09 [-0.24, 0.06]

Table S7 Summary of IO muscle thickness.

Outcome or Subgroup	Studies	Participants	Statistical Method	Effect Estimate
5.1 Muscle thickness	2	112	Mean Difference (IV, Fixed, 95% CI)	-0.02 [-1.14, 1.10]

Table S8 Summary of EO muscle thickness.

Outcome or Subgroup	Studies	Participants	Statistical Method	Effect Estimate
6.1 Muscle Thickness	2	112	Mean Difference (IV, Fixed, 95% CI)	0.10 [-0.64, 0.84]

Table S9 Metaregresion result for abdominal pain.

```
Meta-regression                                          Number of obs  =        4
REML estimate of between-study variance                 tau2           =        0
% residual variation due to heterogeneity               I-squared_res  =    0.00%
Proportion of between-study variance explained          Adj R-squared  =  100.00%
Joint test for all covariates                           Model F(2,1)|  =     1.16
With Knapp-Hartung modification                         Prob > F       =   0.5491
```

MD	Coef.	Std. Err.	t	P>|t|	[95% Conf.	Interval]
age	-.4971302	.4951353	-1.00	0.499	-6.788421	5.79416
length_interv	1.653796	1.397011	1.18	0.447	-16.09691	19.40451
_cons	11.69766	13.25778	0.88	0.540	-156.7584	180.1538

Table S10 Metaregresion result for TrA muscle thickness.

```
Meta-regression                                          Number of obs  =        4
REML estimate of between-study variance                 tau2           =    .4514
% residual variation due to heterogeneity               I-squared_res  =   31.60%
Proportion of between-study variance explained          Adj R-squared  =   93.81%
Joint test for all covariates                           Model F(2,1)   =     7.76
With Knapp-Hartung modification                         Prob > F       =   0.2461
```

MD	Coef.	Std. Err.	t	P>|t|	[95% Conf.	Interval]
Age	-3.738823	1.289004	-2.90	0.211	-20.11718	12.63953
length_interv	11.69134	3.750489	3.12	0.198	-35.96313	59.34582
_cons	93.01212	33.48264	2.78	0.220	-332.4251	518.4494

Higgins JPT, Green S (editors). *Cochrane Handbook for Systematic Reviews of Interventions* Version 5.1.0 [updated March 2011]. The Cochrane Collaboration, 2011. Available from www.handbook.cochrane.org.
